# Infidelity of SARS-CoV Nsp14-Exonuclease Mutant Virus Replication Is Revealed by Complete Genome Sequencing

**DOI:** 10.1371/journal.ppat.1000896

**Published:** 2010-05-06

**Authors:** Lance D. Eckerle, Michelle M. Becker, Rebecca A. Halpin, Kelvin Li, Eli Venter, Xiaotao Lu, Sana Scherbakova, Rachel L. Graham, Ralph S. Baric, Timothy B. Stockwell, David J. Spiro, Mark R. Denison

**Affiliations:** 1 Departments of Pediatrics and Microbiology and Immunology and Elizabeth B. Lamb Center for Pediatric Research, Vanderbilt University Medical Center, Nashville, Tennessee, United States of America; 2 The J. Craig Venter Institute, Rockville, Maryland, United States of America; 3 Department of Epidemiology, University of North Carolina, Chapel Hill, North Carolina, United States of America; 4 Department of Microbiology and Immunology, University of North Carolina, Chapel Hill, North Carolina, United States of America; Fred Hutchinson Cancer Research Center, United States of America

## Abstract

Most RNA viruses lack the mechanisms to recognize and correct mutations that arise during genome replication, resulting in quasispecies diversity that is required for pathogenesis and adaptation. However, it is not known how viruses encoding large viral RNA genomes such as the *Coronaviridae* (26 to 32 kb) balance the requirements for genome stability and quasispecies diversity. Further, the limits of replication infidelity during replication of large RNA genomes and how decreased fidelity impacts virus fitness over time are not known. Our previous work demonstrated that genetic inactivation of the coronavirus exoribonuclease (ExoN) in nonstructural protein 14 (nsp14) of murine hepatitis virus results in a 15-fold decrease in replication fidelity. However, it is not known whether nsp14-ExoN is required for replication fidelity of all coronaviruses, nor the impact of decreased fidelity on genome diversity and fitness during replication and passage. We report here the engineering and recovery of nsp14-ExoN mutant viruses of severe acute respiratory syndrome coronavirus (SARS-CoV) that have stable growth defects and demonstrate a 21-fold increase in mutation frequency during replication in culture. Analysis of complete genome sequences from SARS-ExoN mutant viral clones revealed unique mutation sets in every genome examined from the same round of replication and a total of 100 unique mutations across the genome. Using novel bioinformatic tools and deep sequencing across the full-length genome following 10 population passages in vitro, we demonstrate retention of ExoN mutations and continued increased diversity and mutational load compared to wild-type SARS-CoV. The results define a novel genetic and bioinformatics model for introduction and identification of multi-allelic mutations in replication competent viruses that will be powerful tools for testing the effects of decreased fidelity and increased quasispecies diversity on viral replication, pathogenesis, and evolution.

## Author Summary

Quasispecies diversity is critical to virus fitness, adaptation, and pathogenesis. However, the relationship of fidelity to population diversity is less studied because viral systems with engineered differences in fidelity and bioinformatic methods that robustly measure and compare fidelity and diversity during replication and passage have not been available. Coronaviruses contain the largest and most complex RNA genomes, and encode multiple novel replicase nonstructural proteins (nsps). We previously demonstrated that murine hepatitis virus nsp14-exonuclease (ExoN) activity is required for replication fidelity. In the present report we have generated nsp14-ExoN inactivation mutants of SARS-coronavirus (S-ExoN) that have stable growth defects and dramatically decreased replication fidelity during replication in culture. We used the S-ExoN mutant viruses to define the diversity and stability of the genome during replication and passage, and to test the capacity of deep sequencing to track virus population diversity over time. The experiments demonstrate that viable S-ExoN mutants accumulate large numbers of predominantly unique mutations across the genome, and that increased diversity is continuous over passage. The results establish methods for direct comparison of consensus genome sequences with total population diversity and the impact on viral growth and adaptation.

## Introduction

Observations of RNA viruses have led to the conclusion that they have error-prone polymerases and do not have RNA proofreading functions [1], resulting in low replication fidelity allowing for rapid evolution and adaptation to new environments. Thus, RNA virus populations have been described as quasispecies, a cloud or assemblage of wild-type (WT) and mutant genomes that exist at a mutation-selection equilibrium [2], [3], [4]. Recent studies have shown that virus diversity is essential for adaptive evolution and the capacity to cause disease [5], [6], [7]. Although the extreme genetic variability of RNA viruses is undoubtedly effective for adaptation, it also renders them susceptible to drug-induced population extinction by mutagenesis [2], [8], [9], [10]. Thus, identification and characterization of factors that directly mediate or regulate replication fidelity are critical to understanding RNA virus replication, pathogenesis, and evolution while providing new avenues for intervention and control.

Coronaviruses (CoVs) are positive-sense RNA viruses that include severe acute respiratory syndrome CoV (SARS-CoV). CoVs have the largest genomes of any known RNA viruses, ranging from 26 to 32 kb in size. Assuming that a fixed optimal mutation rate (e.g., one mutation per genome per replication cycle) applies to all RNA viruses irrespective of genome size, then viruses with larger genomes must have higher replication fidelity as they have more opportunity for error in each replication cycle. In this light, it has been proposed that CoVs may have reached the size limit for a replicating RNA molecule and thus must have higher replication fidelity than RNA viruses with smaller genomes. In turn, this has led to the hypothesis that CoVs perform RNA-dependent RNA proofreading during replication [11]. CoVs express up to 16 nonstructural proteins (nsps) that are translated from the input positive-sense RNA genome and processed from polyprotein precursors by two or three viral proteinases. The CoV nsps include proteins shown in vitro to have enzymatic activities consistent with roles in RNA synthesis or modification, including: RNA-dependent RNA polymerase (RdRp; nsp12), RNA primase (nsp8), helicase-NTPase (nsp13), exoribonuclease (ExoN; nsp14), endoribonuclease (EndoU; nsp15), RNA 2′-O-methyltransferase (MT; nsp16), and RNA cap N7-methyltransferase activity (nsp14) [12], [13], [14], [15], [16], [17]. It has been proposed that multiple CoV nsps interact for RNA modifications including error recognition and repair, but this has yet to be tested in defined biochemical systems. The nsp14 exoribonuclease is present in all CoVs, and was predicted based on identification of motifs of the DEDD superfamily of 3′-to-5′ exonucleases, which includes DNA proofreading enzymes [17], [18]. Homologs of nsp14-ExoN are present in the large RNA genomes of toroviruses (28 kb) and roniviruses (26 kb), but not in the smaller genomes of arteriviruses (12–16 kb) nor in any RNA virus family outside the order *Nidovirales* [11]. Recombinant purified SARS-CoV nsp14 has in vitro 3′-to-5′ exoribonuclease but not exodeoxyribonuclease activity, and alanine substitutions of the DEDD residues block activity [16], [19]. We engineered and recovered viable DEDD-to-AADD and DEDD-to-DEDA mutants of murine hepatitis virus (MHV-A59) nsp14-ExoN, and partial genome sequencing demonstrated that ExoN mutant viruses had 15-fold increased accumulation of mutations across the regions analyzed compared to wild-type (WT) MHV during replication in culture [20]. This result, along with the similarity to cellular DNA and RNA proofreading exonucleases suggested that nsp14-ExoN may directly mediate or regulate RNA error prevention or repair. It is unknown whether ExoN is critical for replication fidelity in all nidoviruses possessing nsp14 or homologs (coronaviruses, roniviruses, and torovirurses). Further, it is unknown whether high mutation rates are constitutively maintained during virus population passage and if the rapidly accumulating mutations impair replication fitness. Determination of mutation rates and quasispecies diversity, as well as the impact on virus fitness and pathogenesis, requires analysis of massive genome sequence datasets of replication fidelity mutants and WT viruses during single rounds of replication and during longitudinal passage. This in turn requires new approaches to quantitate and compare diversity from total populations. The advent of next-generation deep sequencing methodologies allows generation of millions or billions of bases in a single experiment, but analysis and interpretation of these data are still a nascent science.

In this study we report the analysis of viable SARS-CoV mutant viruses containing inactivating substitutions in the nsp14-ExoN DEDD motif I (AADD) (S-ExoN1). Comparison of the growth and complete genome sequences of plaque isolates of wild-type SARS-CoV (SARS-WT) and S-ExoN1 demonstrates that all S-ExoN mutant viruses have similar impaired growth compared to SARS-WT, and that inactivation of ExoN results in a 21-fold decrease in replication fidelity compared to SARS-WT. Thus, ExoN is not required for SARS-CoV replication in culture but is required for high-fidelity replication. We performed growth analysis and deep sequencing of SARS-WT and S-ExoN1 population viruses obtained during serial passage in culture, and analyzed massive sequence datasets using newly developed methods for quantifying deviation in a population at each genomic nucleotide position. The results show greater diversity within the S-ExoN1 mutant viruses than SARS-WT at all passages. The profound difference in replication fidelity between S-ExoN1 and SARS-WT viruses, in combination with new quantitative methods for comparison of consensus genomes and total population diversity, will allow direct testing of the impact of decreased fidelity and increased diversity on replication, pathogenesis, adaptation, and fitness of coronaviruses. The experiments described also have identified an extensive library of mutations and mutation sets that can be tested for replication effects, epistatic interactions, and impact on virulence and attenuation.

## Materials and Methods

### Cells and viruses

VeroE6 cells (Vero) were maintained in minimal essential medium (Invitrogen) containing 10% FBS, supplemented with penicillin, streptomycin, and amphotericin B. SARS-CoV Urbani strain (hereafter, SARS-CoV) wild-type and mutant viruses were propagated and assessed by plaque assay on Vero cells. All incubations of cells and virus were at 37°C in a 5% CO_2_ atmosphere. All viral studies were performed in certified BSL3 laboratories and exclusively within biological safety cabinets using protocols for safe study, maintenance, and transfer of SARS-CoV that were reviewed and approved by the Institutional Biosafety Committees of Vanderbilt University.

### Generation of SARS-CoV and ExoN mutant viruses

Viruses containing PCR-generated mutations within the viral coding sequence were produced using the SARS-CoV assembly strategy with the following modifications [21], [22], [23]. The active-site residues of the DEDD ExoN motifs are located in the SARS-CoV cDNA fragment D. The DE residues in ExoN motif 1 were changed to AA using the following primers: GCGTGGATTGGCTTTGCCGTAGCCGGCTGTCATGCAACTAG and CTAGTTGCATGACAGCCGCCTACGGCAAAGCCAATCCACGC. The D residue in ExoN motif 3 was changed to A using the following primers: GGCTAGTTGTGCCGCTATCATGACTAGATGTTTAGCAGTCC and GGACTGCTAAACATCTAGTCATGATAGCGGCACAACTAGCC. Briefly, digested, gel-purified fragments were simultaneously ligated together. Transcription was driven using a T7 mMessage mMachine kit (Ambion), and RNA was electroporated into Vero cells. Virus viability was determined by CPE (cytopathic effect, in this case cell rounding and detachment), and progeny viruses were passaged at low multiplicity of infection (MOI). RNA was recovered from infected cell monolayers using TRIzol (Invitrogen) according to the manufacturer's instructions, and retention of introduced mutations was verified by RT-PCR and DNA sequencing.

### Viral growth and plaque assays

Vero cells were infected at an MOI of 0.1 or 0.01 PFU/cell. After 1 h at 37°C, the inocula were removed, and cells were washed three times and supplemented with prewarmed medium. Samples were taken at each time point and replaced with the same volume of prewarmed medium, then frozen. To determine viral titer, samples were serially diluted, inoculated onto Vero cell monolayers in 6-well plates for 1 h, and overlaid with complete medium plus 1% agar. Plaques were visualized between 48–52 h p.i. by neutral red staining (Sigma) or without staining by using a light box, then counted and titers calculated. For S-ExoN1, we attempted to use P4 virus stocks corresponding to the P3 genomes that were sequenced using the Sanger method. However, storage and freeze-thawing of P3 plaque clone agar plugs resulted in loss of or greatly diminished infectivity of all S-ExoN1 clones used for Sanger sequencing (and ∼50% of all S-ExoN1 clones tested) except for P3 c53. Thus, with the exception of c53, the other four S-ExoN1 clones tested for growth were independent of those sequenced.

### Sanger (dideoxy) sequence analysis of complete viral genomes

Online random number generation software (http://www.randomizer.org) was used to choose which SARS-WT and S-ExoN1 clones (from 60 clones per virus) to sequence using the Sanger (dideoxy) method, resulting in selection of the clones indicated in Figure S1. Vero cells in 25-cm^2^ flasks were infected with virus from solubilized agar plugs for 30–41 h p.i., medium was discarded, and cells were lysed and total cellular RNA extracted using TRIzol reagent. 200 picograms of RNA were subjected to RT-PCR with SARS-CoV-specific primers using a OneStep RT-PCR kit (Qiagen) in 96-well plates and the following thermal cycling conditions: 50°C×30 min, 95°C×15 min, then 5 cycles of 94°C×30 sec, 40°C×30 sec, 72°C×1 min, followed by 30 cycles of 94°C×30 sec, 55°C×30 sec, 72°C×1 min, and a final extension of 72°C×10 min. Entire genomes were amplified as 96 partially overlapping amplicons ranging from 0.4 to 0.8 kbp. Primers were designed using high-throughput primer design software, and primer sequences are available upon request [24]. Each PCR primer included an 18-nt M13 sequence tag (forward primers, TGTAAAACGACGGCCAGT; reverse primers, CAGGAAACAGCTATGACC) for DNA sequencing. For some samples, sequence closure required RT-PCR reactions using additional primer pairs. Amplicons were purified from unincorporated nucleotides and primers either by treatment with exonuclease I and shrimp alkaline phosphatase (USB Corp.) or by electrophoresis and purification from agarose gels. Bulk (uncloned) purified cDNA was directly subjected to automated dideoxy DNA sequencing on a 3730 ABI Sequencer (Applied Biosystems). SeqMan (DNASTAR, Inc.) and the Elvira suite (http://elvira.sourceforge.net) were used for preliminary and final sequence assemblies, respectively. Finished sequences required a minimum of 2-fold amplicon coverage and a total of 4-fold sequence coverage across the entire genome. Note that the requirement for 2-fold amplicon coverage meant that all single-nucleotide polymorphisms (SNPs) were confirmed from independent RT-PCR reactions. GenBank accession numbers are provided in Table S1.

### Mutation rate determinations

The mutation rate (µ) was calculated using the formula µ =  number of substitutions/number of nucleotides sequenced/number of replication cycles. For these calculations, only single-nucleotide substitutions that were unique to individual viral clones were included, while shared mutations and indels were excluded. Based on growth analyses, we estimated that one replication cycle was equivalent to 12 h for SARS-WT and S-ExoN1. The total numbers of cycles from initiation of infection of P1 through RNA harvest at the end of the P3 expansion step were 15 and 24.25 for SARS-WT and S-ExoN1, respectively. The relatively long duration of the initial virus recovery from cDNA clones at P0 (96 or 180 h for SARS-WT and S-ExoN1, respectively) was excluded from determination of number of replication cycles since, assuming that the P1 founder plaque clones originated from a single genome and assuming no back mutation, mutations unique to each clone must have accumulated after P0. An additional assumption was that mutation rates were constant across all passages. Mutation count data for WT and nsp14-ExoN mutants of MHV were from Eckerle et al., 2007 [20]. Data from MHV ExoN1 and ExoN3 (M-ExoN1 and M-ExoN3, previously designated rExoN1 and rExoN3) were pooled (M-ExoN1/3). All SNPs in each M-ExoN1/3 clone were unique to that clone, so for M-ExoN1/3 the rates of accumulation of total and unique substitutions were identical. For calculations of substitutions/genome/cycle, genome lengths were 29,727 nt for SARS-CoV and 31,335 nt for MHV, corresponding to the length of our cloned viral genomes and excluding the length of the 3′-terminal poly(A) tail. MHV genomes were not completely sequenced, but rather mutation count data from regions a-c (defined in [20]) totaling 20,163 nt for each viral clone were pooled for each M-ExoN1/3 and MHV-WT clone.

### Passage series

Flasks of electroporated cells with evidence of CPE were designated as passage 0 (P0) and subjected to a plaque assay. Individual plaques were isolated and expanded on fresh cells, generating stocks that were titered and used to initiate the passage experiment. Three different P1 plaque isolates were used for each virus, SARS-WT (c1, c2, and c3) and S-ExoN1 (c5, c8, and c13), and these were the first three WT clones that were isolated and the S-ExoN1 clones with the lowest clone numbers that produced CPE when transferred to a flask of fresh cells. For SARS-WT, P1 c1 was the same parental clone used to generate the P3 plaque clones subjected to Sanger sequencing, whereas P1 c2 and c3 were unrelated to the P3 clones used for Sanger sequencing. For S-ExoN1, we attempted to use the parental P1 c3 that was used for Sanger sequencing to initiate a passage lineage, however, storage and freeze-thawing of plaque clone agar plugs resulted in loss of infectivity. Thus, P1 c5, c8, and c13 were independent of the P3 clones used for Sanger sequencing. The passage experiment consisted of plating Vero cells in 25-cm^2^ flasks the day before infection. Immediately prior to infection, medium was replaced with fresh medium, and then cells were infected at an MOI of 0.1 PFU/cell. Following 24-h incubation, the supernatant was removed, filtered, aliquoted and frozen. Cells were lysed using TRIzol according to manufacturer's instructions and the lysates frozen. A small sample of each virus from each passage was thawed and the titer determined by plaque assay. Then the appropriate volume of virus was used to initiate the next passage, maintaining an MOI of 0.1 at all passages. SARS-WT and S-ExoN1 clones were each subjected to 20 passages.

### Deep sequence analysis of viral genomes

RNA samples from P1', P5', and P10' in the population passage series were subjected to reverse transcription using Superscript III reverse transcriptase (Invitrogen) and random hexamers at 50°C for 1 h. Entire genomes were amplified from first-strand cDNA by 40 PCR cycles as 13 partially overlapping amplicons ranging from 2.4 to 2.6 kbp (primer sequences available upon request). Thermal cycling conditions were: 95°C×2 min followed by 40 cycles of 95°C×45 sec, 52 or 54°C (depending on the primer pair) ×45 sec, and 72°C×3 min, concluding with a final extension of 72°C×5 min. Amplicons were purified from agarose gels using Wizard SV Gel and Clean-Up kit (Promega) and concentration and purity determined using an ND-1000 spectrophotometer (Nanodrop Technologies, Inc.). For each sample, the 13 amplicons were pooled in equimolar amounts to a final concentration of 125 ng/µl, and 2 µg were used for sample preparation, cluster generation, and multiplexed sequencing using standard commercial kits and protocols for single-end reads (Illumina). For multiplexed sequencing, individual libraries were constructed using genomic adaptor “barcodes” that contain 6-base index sequences and were unique for each sample. Pooled samples were loaded in a single lane of a flow cell and sequencing was performed on a Genome Analyzer II device (Illumina) [25].

### SNP calling in Sanger sequences

Complete Sanger genome sequences were aligned against a reference genome using ClustalW or the Align to Reference tool in MacVector 10.0 (MacVector, Inc.) with default parameters. The two reference genomes used throughout this study were the sequence of the infectious SARS-WT cDNA clone with or without the engineered ExoN1 mutations, as appropriate. SNPs were identified by visual inspection of the multiple sequence alignments. Positions with ambiguous nucleotide codes due to signals for multiple bases at the same position in the experimental Sanger sequences were not considered mutations for this study.

### Mapping of Illumina sequencing reads and identification of SNPs

RazerS software [26] was used to map each 75-base sequencing read uniquely to the genome, initially allowing up to nine mismatches (88% identity) as well as insertions and deletions (indels). Position profiles and SNPs from the read mapping were then generated for each sample. A position profile is defined for each sample as the observed nucleotide distribution at each position along the reference genome, based upon the reads that mapped for that sample. Position profiles for each sample were also generated separately for forward reads and reverse reads, as well as bi-directionally. Before generating the position profiles, several quality filters were applied to the data. First, the last five bases of each read were trimmed to remove low quality bases that tend to be more frequent toward the end of each read. Second, any bases from reads that overlapped with a PCR primer region were trimmed. Third, any base with a quality value <29 was removed from the position profiles. Finally, for the bidirectional position profiles, bases that had <20x coverage in either read direction were excluded. For the determination of SNPs, two additional filters were imposed: 1) at least 20 combined reads from both directions must support the SNP, and 2) the SNP must occur in at least 5% of the reads in each direction. After initial analysis, it was determined that reads with >3 mismatches should be excluded to further reduce the noise level attributable to mapping errors. The list of SNPs identified was unaffected by this additional filter, further supporting the idea that the extra mismatches could be attributed to random mapping errors.

### RMSD calculations and plots

To measure the differences in nucleotide distributions between two samples, the root mean square deviation (RMSD) between them was calculated. Using the position profile that was generated for each sample based on the RazerS read mappings, positional nucleotide distributions were computed and then compared using a modified version of the standard RMSD formula [27]:


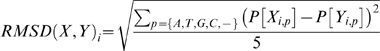
. *P[X_i,p_]* and *P[Y_i,p_]* are the probabilities of nucleotide, *p*, at position *i*, for the sample X and Y, respectively. The nucleotide, *p*, is an element of the nucleotide set {A, T, G, C, –}, where the ‘–’ character, represents a gap that was necessary to align the sequences within the sample against the reference. The position, *i*, ranges from 1 to the length of the gapped alignment, *L*. The number of indels that were necessary to align the sequences in the multiple sequence alignment were also included for each position. The denominator within the square root operator, 5, is the number of possible nucleotides plus the gap.

The minimum RMSD value is 0 when the distributions of nucleotides are identical between the two genomes for a specific position. The maximum value of 0.632 is generated if the allele of one sample is completely different than the second sample, for example, if sample X consists of 100% T's and sample Y consists of 100% G's. Summary RMSD values between two samples were generated by computing the arithmetic mean of each RMSD value for every position along the length of the genome, *L*. The 95% confidence intervals were computed for the summary RMSD values using the bootstrap method with a sample size of *L* and 10,000 trials.

The RMSD plot visualizes the differences between the positional nucleotide distributions of one or more samples and the reference genome. The RMSD plot represents the genomic position along the x-axis and the computed RMSD value for each nucleotide position from the reference along the y-axis. Each colored glyph on the RMSD plot corresponds to a different sample that has been compared to the reference.

### Statistical analyses

Two-tailed Wilcoxon rank-sum tests and Pearson's chi-square tests were performed using STATA 9.1 software (StataCorp, College Station, TX). For all statistical tests a P value of <0.05 was considered significant.

## Results

### SARS-CoV nsp14-ExoN mutants are viable but growth impaired

**Figure 1:**
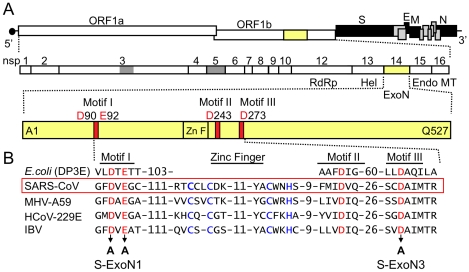
SARS-CoV genome organization and nsp14 exoribonuclease motifs. (A) SARS-CoV genome organization and ORF 1a/b polyprotein expression. The genome is a 29.7-kb positive-sense RNA molecule that is capped (dark circle) and polyadenylated. Genes are indicated for the replicase (ORF 1a and ORF 1b; white), structural proteins [Spike (S), Envelope (E), membrane (M), and nucleocapsid (N) proteins; black], and accessory proteins (light gray). ORF 1b is accessed by ribosomal frameshift in the nsp12 coding sequence. The ORF 1a/b polyprotein is translated directly from input genome RNA and processed into 16 mature nsps by two virus-encoded proteinases (gray). Nsps have predicted or demonstrated activities as described in the text. Hel, helicase; Endo; endoribonuclease; MT, 2′-O-methyltransferase. (B) Organization of nsp14 and partial sequence alignment of representative CoV nsp14 sequences with *Eschericia coli* DNA polymerase III epsilon subunit (DP3E), the proofreading exonuclease subunit of the replicative DNA polymerase (SwissProt P03007). Sequence alignment and GenBank accession numbers for the full-length CoV genomes are as in [20]. Active-site residues of conserved motifs I to III of the DEDD superfamily are indicated in red and by amino acid position in nsp14. A predicted zinc finger domain (Zn F) is located between motifs I and III in the viral sequences, and the predicted zinc-coordinating residues are shown in blue type. Residues replaced with alanine are indicated by black arrows for SARS-CoV mutants S-ExoN1 and S-ExoN3.

**Figure 2:**
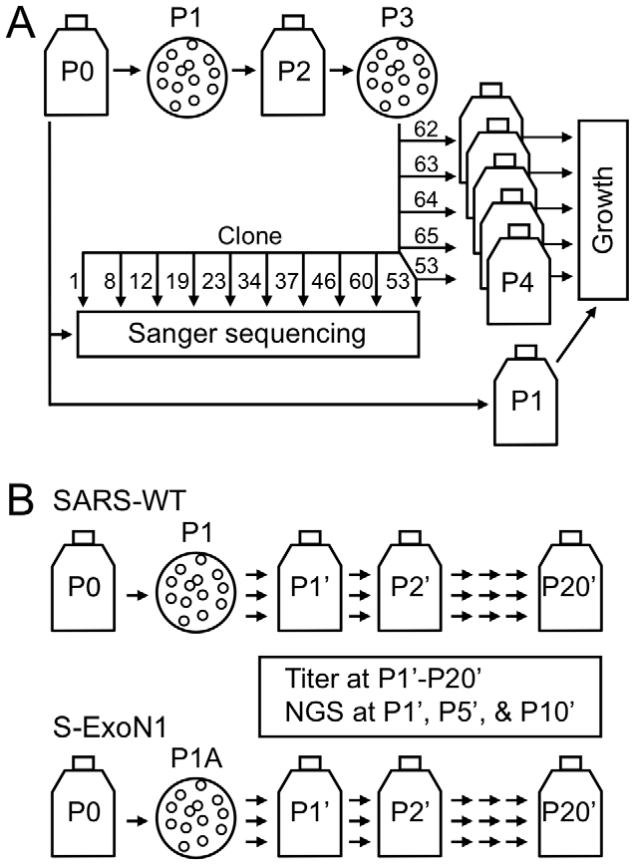
Virus isolation and passage strategy. (A) The strategy used for plaque isolation and passage to obtain stocks used in dideoxy (Sanger) DNA sequencing and viral growth analyses. Flasks indicate population stocks, and circles indicate plaque isolation. A single Passage 1 (P1) plaque clone was the parent of all P3 clones used in complete genome sequence analysis (10 clones) or growth analysis (five clones). Clone numbers are shown. S-ExoN1 P3 clone 53 (c53) was used in both sequence and growth analyses. The scheme shown is for S-ExoN1 but an identical scheme was used for SARS-WT except that clone numbers were different and P3 c21 was used in both sequence and growth analyses. For sequencing, P3 plaque homogenates were expanded on fresh cells and total intracellular RNA was obtained (not shown). Genome sequences were defined as P3, whereas P4 viral stocks were used in growth analyses. (B) Serial population passage. Passage numbers in the serial population passage series are designated with a prime. Three clones of SARS-WT or S-ExoN1 were isolated at P1 or P1A, respectively, and passaged in parallel in the P1'-P20' series. Note that P1A in panel B and P1 in panel A are distinct plaque isolation experiments from the same P0 stock. Viral titers were determined at every passage in the P1'-P20' series and next-generation sequencing (NGS) was performed at P1', P5', and P10' for one clonal lineage each for SARS-WT and S-ExoN1.

To determine whether nsp14-ExoN is required for replication of SARS-CoV, the conserved ExoN active-site residues of motif I, Asp90 and Glu92, or motif III, Asp273, of nsp14 were substituted with alanine in viruses S-ExoN1 and S-ExoN3, respectively (Figure 1, Table 1). The same mutations have been shown to ablate 3′-to-5′ ExoN activity of purified SARS-CoV nsp14 in vitro [16]. All mutations were engineered into the in vitro-assembled full-length cDNA of recombinant SARS-CoV strain Urbani, followed by electroporation of Vero cells with in vitro-transcribed viral genome RNA and nucleocapsid (N) protein transcripts. For both constructs, characteristic SARS-CoV-induced cytopathic effect (CPE) was observed in electroporated cells, and culture medium contained infectious virus as determined by plaque assay. Virus in the culture medium of electroporated cells (passage 0, P0) was used to infect cells for RNA isolation and plaque isolation of viral clones at P1 (Figure 2A). Sequence analysis of RT-PCR products amplified from viral RNA from cells infected with P1 plaque isolates showed that all four S-ExoN1 clones and three of four S-ExoN3 clones retained the engineered mutations. One S-ExoN3 clone isolated from a rare large plaque contained a same-site reversion (GCC-Ala to GAC-Asp) at residue 273, whereas all S-ExoN1 plaques and the majority of S-ExoN3 plaques were small or medium in size compared to the large plaques generated by wild-type SARS-CoV (SARS-WT; data not shown). Retention of motif I mutations was also verified by sequence analysis of two independent P1 stocks of S-ExoN1 that had not been subjected to plaque isolation. Since the motif I mutations appeared more stable than those of motif III, S-ExoN1 was used in all subsequent experiments. Thus, intact ExoN active-site residues in motifs I and III are not required for SARS-CoV replication in culture but are required for efficient plaque development.

**Table 1:** Engineered ExoN mutations.

Virus	Nucleotide substitutions^a^	Codon change	Amino acid substitutions^b^
S-ExoN1	A18238C, T18239C	GAT→GCC	Asp90Ala
	A18244C, G18245C	GAG→GCC	Glu92Ala
S-ExoN3	A18787C, T18788C	GAT→GCC	Asp273Ala

aNucleotide positions refer to the SARS-CoV Urbani strain complete genome sequence (GenBank accession no. AY278741).

bAmino acid positions refer to mature nsp14 sequence (polyprotein 1ab residues 5903-6429).

**Figure 3:**
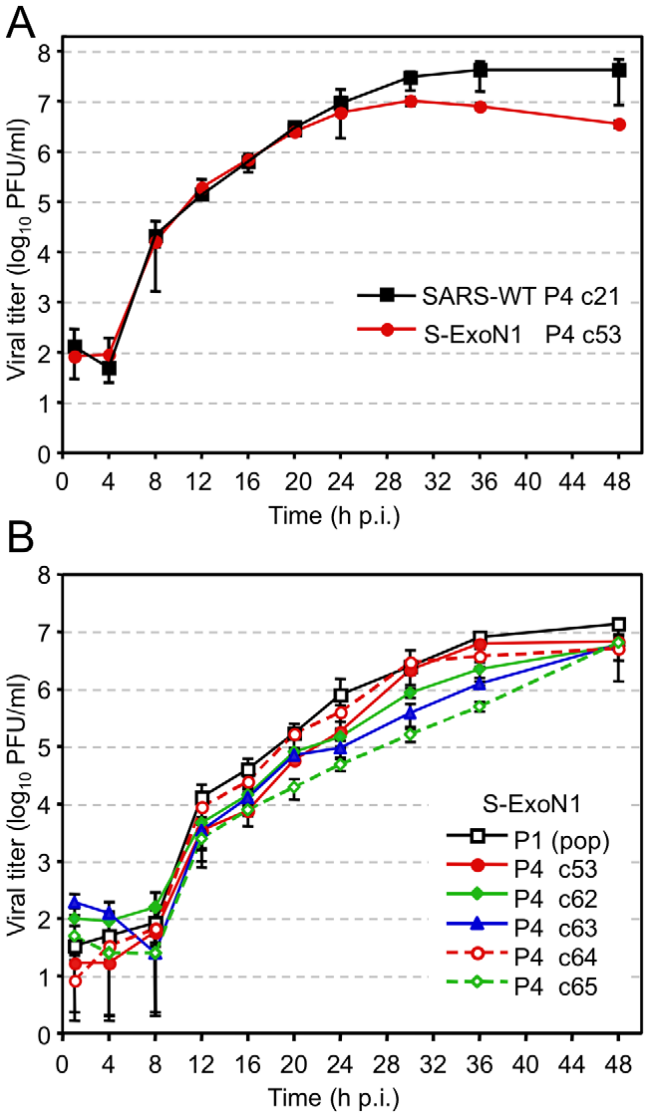
Growth analysis of viral replication. Growth comparisons of SARS-WT and S-ExoN1 viruses (A), and of multiple S-ExoN1 clones and S-ExoN1 population virus (B). (A) Vero cells were infected with SARS-WT P4 c21 and S-ExoN1 P4 c53 viruses at an MOI of 0.1 PFU/cell. (B) Vero cells were infected with P1 population stock of S-ExoN1 or S-ExoN1 P4 clones (c53, c62, c63, c64, and c65) at an MOI of 0.01 PFU/cell. Samples of culture medium were obtained at 1, 4, 8, 12, 16, 20, 24, 30, 36, and 48 h p.i., and viral titers were determined by plaque assay. Mean titers and standard deviations from triplicate infection series are indicated for each time point.

To determine whether substitution of ExoN active-site residues altered viral replication, we performed growth assays in Vero cells using P4 stocks of SARS-WT plaque clones 14 and 21 (c14 and c21) and S-ExoN1 c53 at a multiplicity of infection (MOI) of 0.1 plaque-forming units (PFU) per cell. Growth kinetics of S-ExoN1 and both SARS-WT clones were identical for the first 20 h, after which S-ExoN1 exhibited reduced viral titers compared to SARS-WT. SARS-WT and S-ExoN1 achieved peak viral titers at 30–36 hpi, although this was reduced by 4-fold for S-ExoN1 (Figure 3A). Both SARS-WT clones showed identical growth throughout the experiment (data not shown). These results indicate that inactivation of ExoN results in impaired replication of SARS-CoV.

### SARS-CoV ExoN mutant viruses accumulate nucleotide substitutions across the genome

**Figure 4:**
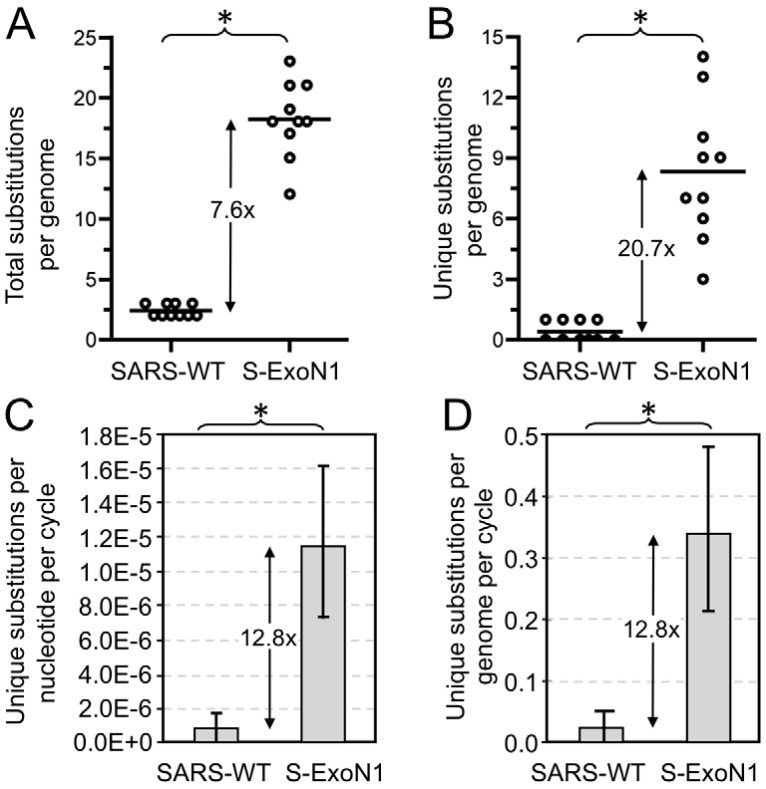
Mutation counts and rates in viral clones. Complete genome sequences were determined for 10 SARS-WT and 10 S-ExoN1 P3 viral clones. Numbers of total (A) or unique (B) non-engineered nucleotide substitutions identified in SARS-WT and S-ExoN1 genomes are indicated. Each circle represents the mutation count value from a single genome sequence and lines indicate mean mutation counts. Rates of accumulation of unique substitutions per replication cycle on a per nucleotide (C) or per genome (D) basis. Mean values are plotted and error bars indicate standard deviations. *, *P*<0.0005; Wilcoxon rank-sum test.

To determine whether S-ExoN1 viruses accumulated more substitutions than SARS-WT, one P1 plaque isolate each of S-ExoN1 and SARS-WT was expanded as a P2 stock, from which multiple P3 plaque isolates were obtained and used to infect new cells for isolation of total RNA (Figure 2A). The entire genome sequences of 10 viral plaque isolates each for S-ExoN1 and SARS-WT were determined from uncloned RT-PCR products (Table S1). Analysis of SARS-WT identified two or three mutations in each genome. In contrast, each of the 10 S-ExoN1 genome sequences contained 12 to 23 non-engineered (secondary) nucleotide substitutions in addition to the four engineered ExoN1 point mutations (Figure 4A). The difference in non-engineered substitution counts between SARS-WT and S-ExoN1 was highly statistically significant (*P*<0.0005, Wilcoxon rank-sum test). Mutations among different clones of SARS-WT and S-ExoN1 were classified as either common to all clones, common to multiple but not all clones, or unique to individual clones. Analysis of the mean substitution counts showed that, compared to SARS-WT genomes, S-ExoN1 genomes contained 7.6-fold more total substitutions (common plus unique) and 20.7-fold more substitutions unique to individual clones (Figure 4, A and B).

**Figure 5:**
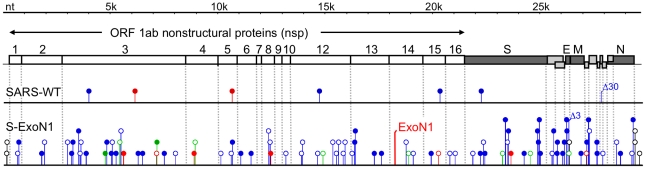
Distribution of mutations across genomes of aggregate viral clones. Combined mutations from 10 SARS-WT and 10 S-ExoN1 P3 viral clones are plotted according to position in the SARS-CoV genome (drawn to scale). Non-engineered mutations are depicted as lollipops and engineered ExoN1 mutations in nsp14 are depicted as a bent vertical line. Red, mutations common to all SARS-WT or all S-ExoN1 clones; green, mutations common to multiple but not all S-ExoN1 clones; blue, mutations unique to one SARS-WT or S-ExoN1 clone. Filled lollipops, nonsynonymous mutations; open lollipops, synonymous mutations; black open lollipops, mutations in non-coding regions. A 30-nt deletion in SARS-WT clone 7 that disrupts ORFs 8a and 8b and a three-nt deletion identified in S-ExoN1 clone 46 in ORF E are indicated above the lollipops by Δ30 and Δ3, respectively. All mutations identified in SARS-WT genomes occurred at nucleotide positions distinct from those in S-ExoN1 genomes. White boxes, nsp domains encoded by ORF1. For simplicity and because no mutations were detected in nsp11, a predicted 17-aa polypeptide that partly overlaps with the amino-terminus of nsp12, nsp11 is not shown. Dark gray boxes, ORFs encoding structural proteins: S, Spike attachment glycoprotein; E, Envelope protein; M, Membrane protein; N, Nucleocapsid protein. Light gray boxes, ORFs encoding group-specific (accessory) proteins.

Within the ∼300 kb of total sequenced nucleotides for both S-ExoN1 and SARS-WT, a total of 100 different non-engineered mutations (99 substitutions and one deletion) were identified in the 10 S-ExoN1 genomes, whereas only seven different non-engineered mutations (six substitutions and one deletion) were identified in the 10 SARS-WT genomes (Figure 5, Table 2). For every mutation, the genomic position, level of conservation among clones, and predicted coding effect are shown in Figure S1 and Tables S2 and S3. All mutations identified in S-ExoN1 genomes occurred at nucleotide positions distinct from those in SARS-WT genomes. For SARS-WT, the C6122T and A10646G mutations found in all P3 clones also were detected in P0 population virus as polymorphisms with the wild-type reference nucleotide. None of the secondary mutations in S-ExoN1 clones were identified in P0 population virus, suggesting that the mutations were generated during passage and fixed in the population by plaque isolation. Two mutations in the SARS-WT group and 16 mutations in the S-ExoN1 group were identified in multiple genomes within each respective group. The mutations identified in all S-ExoN1 clones or all SARS-WT clones were likely fixed in the single P1 parent clone of each group, as was shown by detection of the same mutations in the parental SARS-WT P1 c1 (Table S4). Mutations identified in multiple but not all S-ExoN1 clones likely arose during P1 plaque outgrowth or P2 expansion, followed by accumulation of unique mutations in each clone after isolation of P3 plaques.

**Table 2:** Mutation types and frequencies.

			Non-redundant mutations^b^							
Virus	No. of clones	Total nt sequenced^a^	NS	Syn	NCR	Indel	Total	NS:Syn	Ts:Tv^c^	Substitution frequency^d^
SARS-WT	10	296,468	6	0	0	1	7	n.a.	1.0	2.02E-05
S-ExoN1	10	296,471	49	44	6	1	100	1.1	5.2	3.34E-04

ant, nucleotides. Values are sums for all P3 clones of each virus.

bValues are derived from the aggregate of all P3 clones for each virus and include shared and unique non-engineered mutations. Each shared mutation was counted only once. Substitutions were categorized as NS, nonsynonymous; Syn, synonymous; or NCR, non-coding region. Indel, insertion or deletion. A T25783A mutation in S-ExoN1 clone 34, which is Syn in ORF 3a but NS in ORF 3b, was categorized as NS here and in Figure 5.

cTs, transition; Tv, transversion.

dSubstitution frequency was calculated by dividing the sum of NS, Syn, and NCR mutations by the total number of nucleotides sequenced.

The mutations in S-ExoN1 were distributed across the genome (Figure 5). To determine whether the distribution of mutations was non-random, the 29,727-nt genome was arbitrarily divided into equal thirds with division points between nt 9909/9910 and nt 19,818/19,819. The 5′, middle, and 3′ thirds contained 29, 26, and 44 mutations, respectively, from the aggregate clones, but the differences were not significant (*P* = 0.06, Pearson's chi-square test). When the genome was instead divided in half, no statistical evidence was observed for non-random distribution between the 5′ and 3′ halves of the genome (41 and 59 mutations, respectively; *P* = 0.072). Distribution analyses could not be performed on SARS-WT due to insufficient numbers of mutations in the 10 clones. While the combination of mutations from different genomes and division of the genome for analysis both support the hypothesis that the mutations are random, they do not constitute proof, since epistatic interactions could potentially influence the unique mutation sets observed in each clone. Total non-redundant substitution frequencies (where each shared mutation was counted only once) were 16.5-fold higher in S-ExoN1 than SARS-WT genomes (3.34E-04 and 2.02E-05 substitutions per nucleotide, respectively, Table 2). The seven substitutions identified in SARS-WT genomes were nonsynonymous (NS), whereas the S-ExoN1 genomes had similar numbers of NS and synonymous (Syn) substitutions as well as six mutations in non-coding regions. Analysis of all coding regions of S-ExoN1 genomes showed that coding regions for nsp3, nsp4, nsp8, and Spike glycoprotein regions had one or more NS mutations in every S-ExoN1 genome (Figure S1B). For Spike alone the 20 sequenced SARS-WT and S-ExoN1 genomes resulted in identification of 11 NS mutations in Spike that were tolerated for replication and had not been previously reported. Coding changes also resulted in disruptions or extensions of ORFs 8a/b in one SARS-WT clone and ORFs 3b and 7a/b in three different S-ExoN1 clones (Tables S2 and S3). S-ExoN1 P3 c46 had a deletion that removed the carboxy-terminal residue Val76 of the envelope (E) protein. Overall, the sequencing data show that engineered S-ExoN1 mutations result in a mutator phenotype similar to that observed for MHV ExoN mutants. These results establish that ExoN activity is required for replication fidelity of divergent coronaviruses.

To determine how the mutation rates compared between this SARS-CoV study and our previous MHV study, we calculated mutation rates for S-ExoN1 and SARS-WT and compared them with those for M-ExoN1, M-ExoN3 and MHV-WT [20]. For these calculations, numbers of unique substitutions in individual viral clones were used, whereas shared substitutions and indels were excluded. Assumptions include a constant mutation rate at all passages, no back mutations, and a 12-h replication cycle for S-ExoN1 and SARS-WT. S-ExoN1 had a 13-fold (*P*<0.0005, Wilcoxon rank-sum test) higher substitution rate than SARS-WT (Figure 4C). This difference is less than the 21-fold difference in substitution frequencies because S-ExoN1 viruses were grown for longer durations than SARS-WT at the P2 and P3 steps. For comparison, M-ExoN1/3 had a 13-fold (*P*<0.01) higher substitution rate that MHV-WT. This value is similar to the reported 15-fold difference in substitution frequencies because although M-ExoN1/3 grew more slowly than MHV-WT, the total number of replication cycles for both viruses was identical. Although the rate for SARS-WT was 2.8-fold lower than MHV-WT, this difference was not statistically significant.

S-ExoN1 had a 2.9-fold lower (*P*<0.005) substitution rate than M-ExoN1/3. The reason for this difference is unclear. One possibility is that SARS-CoV may have additional or more efficient mutation correction mechanisms. An alternative explanation is that the SARS-CoV study was not designed to determine mutation rates. For example, durations of S-ExoN1 and SARS-WT infections used for rate determination were estimates since exact durations were not recorded. It is unclear if differences in the virus passage and plaque isolation protocols for the two studies contribute to differences in mutation rates, and if so, to what extent. Yet another possibility is that approximately one-third of each MHV genome was not sequenced, so the MHV mutation rates may not be representative of the entire genome.

### Effect of ExoN mutant virus mutator phenotype and mutations on growth during infection and passage in culture

To determine whether the secondary mutations generated during S-ExoN1 replication alter growth, we compared the replication kinetics of S-ExoN1 P1 uncloned population virus with five S-ExoN1 P4 plaque isolates, including the completely sequenced c53. S-ExoN1 P1 population virus and all five P4 viral clones grew with similar kinetics and achieved equivalent peak viral titers in Vero cells by 48 h p.i. (Figure 3B). From 12–36 h p.i., titers of all P4 clones were equivalent or modestly reduced compared to P1 virus. These results suggest that S-ExoN1 replication in Vero cells is unaffected by the non-engineered mutations in viral clones or at least the cumulative effect of all mutations in each clone (mutation set).

**Figure 6:**
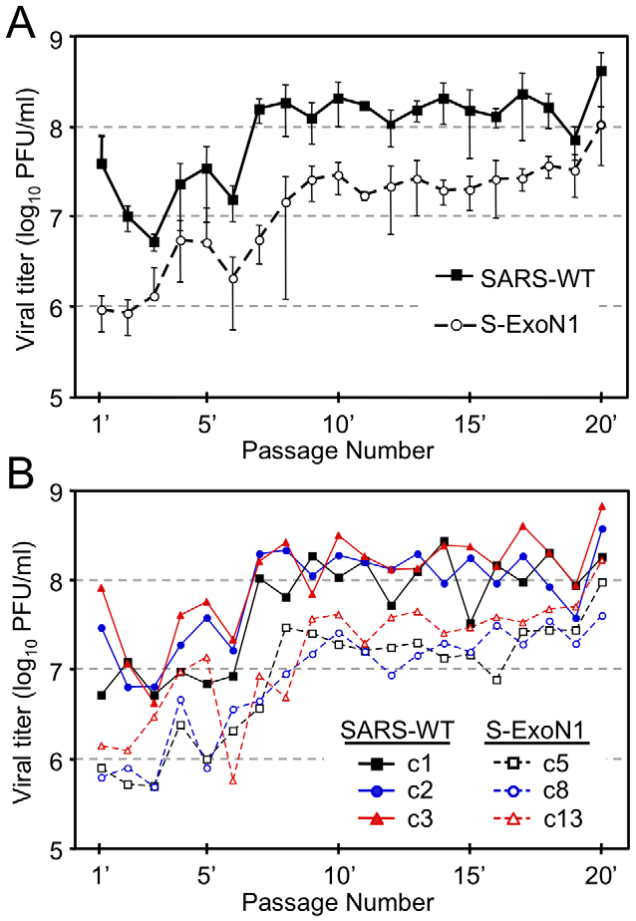
SARS-WT and S-ExoN1 titers across 20 population passages. (A) Mean viral titers of three clones each of SARS-WT and S-ExoN1 at passages 1'-20'. Error bars indicate standard deviations. (B) Viral titers of individual clones of SARS-WT (closed symbols, solid lines) and S-ExoN1 (open symbols, dashed lines) at passages 1'-20'. At each passage Vero cells were infected at an MOI of 0.1 PFU/cell. Samples of culture medium were collected at 24 h p.i., titered by plaque assay at each passage, and transferred to naive cells.

To determine whether replication of S-ExoN1 is stable over time, we passaged three plaque isolates each of SARS-WT and S-ExoN1 20 times (P1'-P20') at the population level in Vero cells at an MOI of 0.1 PFU/cell for 24 h (Figures 2B and 6). Overall, mean titers of the three clones for both SARS-WT and S-ExoN1 increased over passage, and the increase was more substantial for S-ExoN1 (∼2 log_10_) compared to SARS-WT (1 log_10_) (Figure 6A). Mean titers showed little change from P7'-P18' for SARS-WT and P9'-P19' for S-ExoN1. Titers of individual S-ExoN1 and SARS-WT clones were largely non-overlapping, and all six clones showed maximal or near-maximal titer at P20' (Figure 6B). These results demonstrate that population passage of both SARS-WT and S-ExoN1 selects for increased growth during population passage in Vero cell culture, and suggest the possibility that growth adaptation is independent of the ExoN mutator phenotype. We recognize that while growth assays are a good measure of relative replication and virus production, they cannot be used to compare virus fitness, which would require competitive coinfection and passage [28], [29]. Since S-ExoN mutants generate mutations in all identified genomes, we would predict that any competitive fitness experiment would be influenced - either positively or negatively – by the decreased fidelity and increased diversity of S-ExoN mutants arising during competitive passage. Thus, fitness experiments of S-ExoN mutator mutants would need to take into account the balance between accelerated evolution and accumulation of large numbers of random mutations in individual genomes and the total virus population. Testing of fitness for individual mutations or groups of mutation in virus clones (mutation sets) or cumulative mutations in a population therefore would require recapitulation of the mutation(s) or mutation sets in the background of WT ExoN, in order to have a more stable genetic background during replication with fitness and selective pressure determined by the introduced mutations and allowing direct comparison with SARS-WT.

### Deep genome sequencing reveals retention of S-ExoN1 engineered substitutions and increased mutation frequencies across the S-ExoN1 genome during population passage

To determine whether genetic diversity of S-ExoN1 viruses increases over passage and whether the engineered ExoN1 mutations are retained, we employed massively parallel deep (Illumina) DNA sequence analysis on P1', P5', and P10' of S-ExoN1 c5 and SARS-WT c1 from the serial passage experiment described above. Total intracellular RNA from infected monolayers was used for reverse transcription and amplification into 13 overlapping amplicons that were processed and subjected to deep sequence analysis [25]. The mean sequence coverage depth ranged from 486 to 1004 reads per nucleotide for each of the six samples, with greatest coverage across regions of amplicon overlap, as reported by others [30] (Figure S2). For S-ExoN1 c5 P1', P5', and P10', 98.6–99.7% of sequence reads across nsp14 codons 90 and 92 contained the four engineered nucleotide substitutions resulting in alanine substitutions. The small fraction of reads that did not contain all four engineered mutations could be explained by the reported ∼1% error frequency of Illumina sequencing [31]. This result demonstrates that the engineered ExoN1 mutations are stably maintained through 10 passages and that same-site reversion was not responsible for the observed increased viral titers during passage.

**Figure 7:**
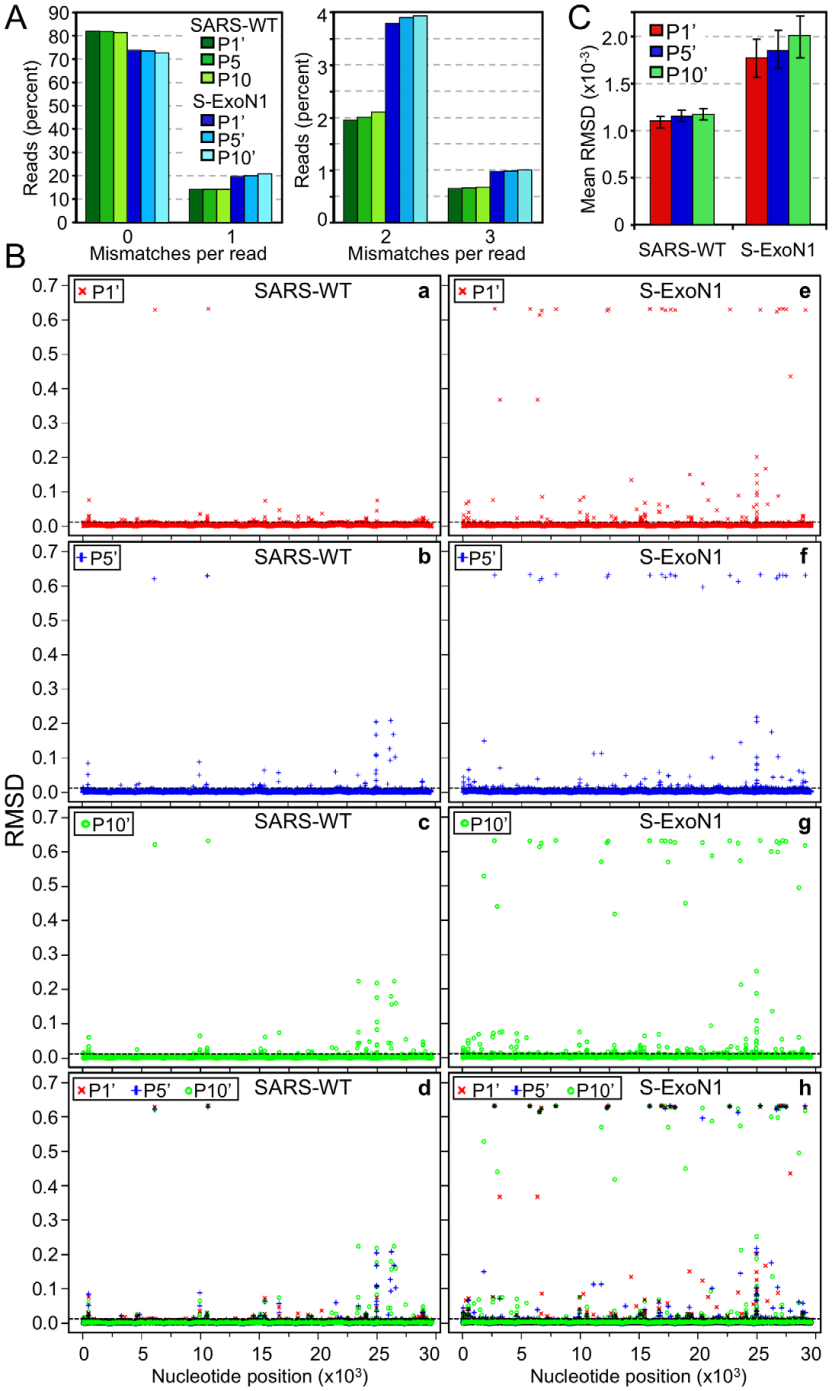
Genetic diversity of SARS-WT and S-ExoN1 from P1', P5', and P10'. (A) The percentage of reads within each sample that have 0-1 (left) or 2–3 (right) mismatches compared to the corresponding reference sequence are shown. (B) RMSD plots. For each SARS-WT sample (a–d) or S-ExoN1 sample (e–h) the root mean squared deviation (RMSD) from the corresponding reference sequence is plotted for each nucleotide position in the genome. Plots are for P1' (a, e), P5' (b, f), P10' (c, g), or combined P1', P5', and P10' (d, h). The maximal RMSD value of 0.632 indicates completely different single-allelic distributions between experimental and reference sequences whereas values ≤0.0125 (dashed line) were considered background. (C) Summary RMSD values between SARS-WT P1', P5', or P10' or S-ExoN1 P1', P5', or P10' and reference sequences were calculated by computing the arithmetic mean of each RMSD value for every position in the genome. The 95% confidence intervals are indicated by error bars.

To compare global diversity of SARS-WT and S-ExoN1, we determined the percentage of reads with 0–3 mismatches at P1', P5', and P10'. A higher percentage of reads from S-ExoN1 had one, two, or three mismatches, whereas a higher percentage of reads from SARS-WT had no mismatches (Figure 7A). No short insertions or deletions were confirmed in at least 20 combined sequence reads of both directions from any of the six samples, and due to the short read lengths, we did not attempt to identify long insertions or deletions. These results indicate that S-ExoN1 had substantially greater genetic diversity than SARS-WT. To determine and compare the distributions of substitutions across the genome, the root mean square deviation (RMSD) between each sample and a SARS-WT or S-ExoN1 reference sequence, as appropriate, was calculated and plotted for every nucleotide position at P1', P5', and P10'. In this analysis, the maximum RMSD value of 0.632 indicates completely distinct single allelic distributions, and values below 0.0125 were considered background. Each of the S-ExoN1 samples had a larger number of points with RMSD values above background than each of the SARS-WT samples, and this was particularly evident for points with values of 0.3 through 0.632 (Figure 7B). These results and the increased mean RMSD of each S-ExoN1 compared to each SARS-WT sample (Figure 7C) indicate that the three S-ExoN1 samples have greater diversity than the three SARS-WT samples relative to the reference sequences at P1', P5', and P10'. These results are consistent with decreased replication fidelity of S-ExoN1. RMSD analyses also demonstrated a trend of increased diversity over passage for both viruses although the differences between passages were not statistically significant and not as great as those between S-ExoN1 and SARS-WT.

We next compared the numbers and patterns of points with near-maximal to maximal RMSD values (RMSD = 0.5 to 0.625) in the RMSD plots and catalogued all dominant single-nucleotide polymorphisms (SNPs) identified in >5% of relevant reads and plotted their frequencies (Tables S5 and S6, Figure S3). Analysis of numbers of points with RMSD values of 0.5 through 0.632 corresponding to SNP frequencies of >0.8, showed S-ExoN1 P1', P5', and P10' had 20, 22, and 29 SNPs in this category, respectively. All of the SNPs with high RMSD values from P1' were carried through in P5' and P10', and those new in P5' were carried through in P10' also such that S-ExoN1 P5' gained two new SNPs relative to P1', and P10' gained seven new SNPs relative to P5', again considering only those that met the above criteria. For SARS-WT, the two positions with maximal RMSD values were present at all three passages analyzed, and all other SNPs were present at much lower frequencies. These results suggest that both substitutions present in the entire SARS-WT population and the majority of those in the entire S-ExoN1 population were already fixed in the viral stocks used to initiate the passage series, likely during plaque isolation. This interpretation is supported by the fact that the two mutations present in >96% of reads spanning those positions from SARS-WT P1', P5', and P10' were identified by Sanger sequencing in all 10 SARS-WT P3 genomes and the parental P1 c1 genome (Table S2). SNPs identified by deep sequencing in S-ExoN1 P1', P5', and P10' were not identified by Sanger sequencing of P3 genomes because for S-ExoN1 independent parental clones were used to derive the samples for Sanger sequencing and to initiate the population passage series (Figure 2). The results further suggest that the few points with maximal or near-maximal RMSD values unique to S-ExoN1 P10' are candidate adaptive mutations. In contrast, no unique individual SNPs were detected in >40% of reads for SARS-WT P10', suggesting that increases in titer observed over passage were due to different mutations in different genomes such that no single mutant genotype achieved dominance in the population.

## Discussion

In this report, we demonstrate that ExoN active-site motifs are not required for SARS-CoV replication, but that inactivation of ExoN results in impaired growth and a high-level mutator phenotype that is maintained during virus passage in culture. Together with our previous study of MHV nsp14-ExoN mutants, the results support the theory that nsp14-ExoN serves to maintain high-level replication fidelity, and likely serves a role in RNA-dependent error recognition, prevention, and repair (i.e., proofreading) in all known viruses with RNA genomes that encode nsp14 homologs: coronaviruses, roniviruses, and toroviruses. The formal demonstration of RNA proofreading in coronaviruses remains a challenge since the coronaviruses express 15 or 16 replicase/transcriptase nsps including RdRp (nsp12), RNA primase (nsp8), endonuclease (nsp15), and RNA cap methyltransferase (nsp14, nsp16) activities in addition to nsp14 ExoN activity [14]. Immunofluorescence, immuno-EM, and protein association studies indicate co-localization and interactions among multiple replicase nsps in membrane-bound complexes, and thus nsp14-ExoN likely functions cooperatively within a multi-protein complex for RNA initiation and elongation of RNA synthesis as well as nucleotide recognition, excision, and repair [13], [32], [33]. Recent development of transient in vitro replication systems from lysed SARS-CoV infected cells may provide an opportunity to address these questions by direct comparison of SARS-WT and S-ExoN1 viruses [34].

Microbiological organisms are subjected to environmental conditions that may change drastically from host to host or within a host. When this is considered alongside the requirement to balance genome stability with diversity to maintain replicative fitness, it becomes logical to propose that mechanisms for regulating replication fidelity might have evolved. This assumption is borne out in organisms such as *Streptococcus pyogenes* SF370, which exhibits a growth-phase-dependent 100-fold variation in replication fidelity due to reiterative excision of a prophage (SF370.4) within the mismatch repair (MMR) gene cassette encoding mutS and mutL [35]. SF370.4 is integrated between mutS and mutL during stationary phase when resources are limited, blocking expression of mutL and resulting in loss of MMR and a profound mutator phenotype. In contrast, prophage SF370.4 is excised as an episome during exponential phase growth allowing mutL expression, intact MMR, and increased fidelity. The observed tolerance in S-ExoN1 replication for 21-fold changes in replication fidelity and associated increased mutation load supports the theory that some RNA viruses may have evolved similar strategies for regulation of fidelity. Such a system could contribute to the stability of genome sequence during replication under non-selective conditions but allow for rapid adaptation to deleterious mutations or new environments. The expression of multiple distinct proteins from the coronavirus genome that are known or predicted to be involved in RNA synthesis or modification also might support a regulated system for variable replication fidelity based on virus protein precursor and mature forms, protein concentrations, and altered virus-host protein interactions. Although this model is speculative, it is consistent with our results demonstrating increased diversity in S-ExoN1 genomes and viral clones during single infections, but lack of significant increase in diversity of S-ExoN1 populations over 10 passages. The nsp14-ExoN mutator viruses and new bioinformatics analyses will allow us to determine whether SARS-WT and S-ExoN mutant viruses demonstrate changes in fidelity under a variety of conditions including responses to different cell types, mutagens, antibodies, antivirals, and during replication in vivo. The mutator phenotype associated with engineered ExoN mutations constitutes a unique property with unclear consequences for adaptation and pathogenesis in animals. Recently, we have recovered viable ExoN mutants in the virulent mouse-adapted SARS-CoV – MA15 background (MA-ExoN) [36]. Experiments in progress will test whether MA-ExoN has altered genetic diversity, virulence, and pathogenesis in mice in comparison with MA15, SARS-WT, and S-ExoN1.

### Analysis of viral genetic diversity using massive sequence datasets

The extent of genetic diversity in RNA virus populations has most often been analyzed by sequencing a small number of genomes at low coverage [37], [38], [39] or a small region at high coverage [40], [41], [42]. The former approach has the limitation of a lack of resolution and cannot detect variants present at low proportions. The latter approach has the limitation of being narrowly focused and, since mutation frequencies may vary depending on region, extrapolation to the entire genome can be misleading. To circumvent the limitations of these approaches, we utilized both dideoxy and deep sequencing across the entire genome of a number of SARS-CoVs. In addition, these viruses were generated in a controlled environment over a number of passages at identical MOI, to examine diversity across different virus isolates from the same passage or within the population over serial passage. Both SARS-WT and S-ExoN1 virus titers increased over passage in cell culture, indicating that both viruses underwent some level of adaptation to growth in Vero cells but still maintained differences in titer. However, passage alone did not induce the increased mutation frequency observed in S-ExoN1 viruses, as SARS-WT was subjected to the same conditions and exhibited few changes in the viral genome. The analyses allowed comparison of the capacity of Sanger and deep sequencing to define population diversity and differences in fidelity. To achieve this goal, it was necessary to develop new methods for analysis of massive datasets generated by deep sequencing of SARS-WT and S-ExoN1 viral RNA. The RMSD plots in this report constitute a novel approach to graphically visualize total diversity across a genome. The readout can be viewed as a diversity map of differences at every position. The RMSD diversity plots can be visualized and analyzed at different resolutions. For example, adaptive mutations that are selected early and that become dominant in the population may be visualized at full resolution, while minor or emerging mutations may be identified or predicted by low-level changes over time by analysis of a subsection of the RMSD plots for multiple passages. We anticipate that this approach could be applied to evaluate deep sequence data from any RNA/DNA virus both in vitro and in vivo.

Using these methods, we found that S-ExoN1 population viruses had greater diversity than SARS-WT at each of the passages analyzed (P1', P5', and P10'), consistent with decreased replication fidelity indicated by Sanger sequencing of individual virus isolates. In contrast, diversity differences between passages of S-ExoN1 were small and not statistically significant, which we speculate may be because most mutations did not confer sufficient selective advantage to accumulate in substantial proportions of the population during limited passage. Some SNPs increased or decreased in frequency over passage. Those that increased may confer replication or infectivity advantages, whereas those that decreased may confer selective disadvantages. A lack of increase in mean RMSD over passage could theoretically be caused if a second-site mutation which complemented the fidelity defect arose at or before P1' of the passage series. However, it is highly unlikely that any of the SNPs directly restored ExoN activity since titer differences between S-ExoN1 and WT were similar throughout the passage series. Nonetheless, one or more non-engineered SNPs may have conferred increased titers by improving some other aspect of the viral life cycle. Alternatively, other mechanisms could function to limit diversity during selection for increased replication, such as recombination repair, rapid loss of highly deleterious or lethal mutations, or other replicase proteins that independently or cooperatively mediate error recognition and repair with nsp14.

### Functions of nsp14 other than replication fidelity

In the report that first demonstrated ExoN activity from purified SARS-CoV nsp14, it was found that substitution of ExoN active-site residues in the full-length human coronavirus 229E (HCoV-229E) genome prevented recovery of infectious virus, while allowing limited genome replication and subgenomic RNA synthesis [16]. In our study of MHV ExoN mutants we demonstrated that those viruses had more severe growth impairments, including a prolonged eclipse phase and larger reductions in peak titers than S-ExoN1 [20]. Several possibilities may explain the differences in the three viral systems. First, it is possible that active-site substitutions result in less complete inactivation of SARS-CoV ExoN activity. Minskaia et al. reported low-level residual ExoN activity from purified SARS-CoV nsp14 with active-site substitutions [16], but in vitro ExoN assays have yet to be developed using nsp14 from other CoVs. Second, unique SARS-CoV accessory proteins or interacting cellular proteins may mask or compensate for defects caused by ExoN inactivation. Reports by us and others indicate that nsp14-ExoN is involved in viral RNA synthesis (see below), so a third possibility is that RdRp activity from SARS-CoV may be more robust than that of the other viruses and compensates for an RNA synthesis defect associated with inactive or less active nsp14-ExoN.

S-ExoN1 viruses maintained a growth defect relative to SARS-WT in all passages examined, suggesting that ExoN has a non-redundant function in coronaviruses. The primary sequence conservation of nsp14 between SARS-CoV and MHV (57% identical and 72% similar across the entire protein) suggest that the function of ExoN responsible for the growth defects is the same in both viruses. CoV ExoN is required for efficient viral RNA synthesis as shown for MHV ExoN mutant viruses [20], a SARS-CoV replicon harboring a deletion of nsp14 or substitution of the ExoN motif II invariant glutamate [43], and HCoV-229E ExoN mutant genomes in electroporated cells [16]. Thus, we speculate that S-ExoN1 has impaired RNA synthesis (genome replication, subgenomic RNA transcription, or both), which results in the observed growth defects. Purified SARS-CoV nsp14 recently has been shown to have RNA cap N7-methyltransferase activity that is blocked by a D331A substitution, probably by preventing binding of the methyl donor S-adenosylmethionine [14]. The D331A substitution reduces levels of transcripts from a SARS-CoV replicon but has no effect on ExoN activity of purified nsp14. A D90A+E92A double substitution in ExoN motif I does not affect methyltransferase activity in vitro. Thus, ExoN1 mutant viruses are likely defective in a step other than cap formation. Another possibility is that the increased mutational load due to impaired fidelity is directly responsible for the growth defects. Finally, it is possible that the engineered amino acid substitutions alter interactions between nsp14 and viral or cellular proteins important for efficient growth.

### Potential implications for coronavirus resistance to mutagens

Proposed treatment regimens for SARS include ribavirin, a nucleoside analog that induces lethal mutagenesis of other RNA viruses such as poliovirus, foot and mouth disease virus, hepatitis C virus, and Hantaan virus [8], [9], [44], [45]. Of interest, a ribavirin-resistant RdRp mutant of poliovirus has been identified that has increased replication fidelity [46], [47], [48]. The high replication fidelity of SARS-CoV in cell culture in this study and the lower-than-expected mutation rate of SARS-CoV in the late stage of the 2002–2003 epidemic [49], [50] raise the possibility that drug-induced lethal mutagenesis therapies may be less effective against coronaviruses than other RNA viruses. A possible recalcitrance of coronaviruses to lethal mutagenesis also is suggested by our demonstration that at least in cell culture S-ExoN1 tolerates 21-fold increase in substitution frequency, whereas a two-to-six-fold increase in mutation frequency was sufficient to cause lethal mutagenesis of poliovirus in cell culture [9], [51]. Based on our present results, we suggest the possibility that inhibitors of nsp14-ExoN activity could decrease replication fidelity, and in simultaneous or sequential combination with an RNA mutagen, render SARS-CoV more susceptible to drug-induced lethal mutagenesis [52]. The high conservation of nsp14-ExoN sequences among coronaviruses and demonstration of mutator phenotypes in both MHV and SARS-CoV suggest that such approaches might be successful across coronaviruses, including viruses newly identified and possibly emerging from bats to other animals [53].

### Replication fidelity and mutation rates

The results in this study with SARS-CoV and in the previously reported MHV ExoN mutants, describe the greatest tolerated change in replication fidelity in infectious viruses reported for an animal RNA virus. In addition, we predict that results for S-ExoN1 likely under-represent the total number of nucleotide misincorporations that occur during RNA replication, for several experimental and biological reasons. First, these studies were performed using infectious viruses, so all mutations must be compatible with virus viability. Second, this subset of mutations is further restricted by selection of those present in the most rapidly replicating genomes in each population that result in visible plaques or dominant populations. Third, as evidenced by the increased virus titer during passage of both SARS-WT and S-ExoN1, adaptive change for increased growth, and possibly fitness, can be selected even in the setting of 21-fold decreased replication fidelity and accumulation of mutations. Finally, calculation of comparative mutation rates between SARS-WT and S-ExoN1 is complicated by differences in the number replication cycles resulting from growth defects of S-ExoN1, and consequent increased time required for plaque development or productive passage and cytopathic effect. Thus, we interpret our results in terms of overall productive viral replication fidelity, rather than intrinsic fidelity of the RNA replication machinery. Our ability to demonstrate decreased fidelity in plaque isolates and to perform deep sequencing on total infected cell RNA indicates that we will be able to use this system to assess total mutations under defined and reproducible conditions of in vitro and in vivo selection, and thereby closely estimate mutation rates. However, direct assessments of fidelity and mutation rates of the viral replication machinery likely will require well-defined in vitro replication systems or replicon studies, ideally where the replicase proteins are expressed independently from an RNA species distinct from the RNA template under investigation.

With those caveats we estimated mutation rates of SARS-WT and S-ExoN1 and compared these with our published results of MHV-ExoN. The S-ExoN1 and M-ExoN1/3 mutation rates of 1.2×10^-5^ and 3.3×10^-5^ substitutions per nucleotide per replication cycle, respectively, from this study are within the range reported for other RNA viruses [1], [51]. In contrast, SARS-WT and MHV-WT mutation rates of 9.0×10^-7^ and 2.5×10^-6^ substitutions per nucleotide per replication cycle, respectively, calculated here are below the expected range of ∼10^-3^ to 10^-5^ reported for other RNA viruses and more similar to the rates for some small ssDNA viruses [54], [55]. While we acknowledge that it is difficult to compare across experimental systems that use different protocols and assays, our data are consistent with a role for nsp14 in RNA proofreading or repair.

### Insights from the mutations in S-ExoN1 mutant viruses

This study revealed several non-synonymous mutations that have not been previously reported and that have little or no effect on viral growth in culture. The most dramatic of these are deletions, nonsense mutations, and missense mutations that altered 5-117 codons in ORFs 3b, 7a, 7b, 8a, and 8b, consistent with previous reports demonstrating that the corresponding proteins are dispensable for growth in culture and in animals [56], [57]. As predicted, no mutations were identified in RNA sequences that are critical for subgenomic RNA transcription since efficient transcription of each subgenomic RNA requires annealing of leader and body transcription regulating sequences [58], [59], [60], [61]. Interestingly, a T-to-C substitution was detected at nucleotide 26,354 immediately following the RNA5 (M gene) transcription regulating sequence (nt 26,348-26,353) in S-ExoN1 P3 clone 8 by Sanger sequencing (Table S3) and in 7% of deep sequence reads across that position in S-ExoN1 P5' (Figure S3). It is unknown if T26354C alters levels of RNA5 or M protein or viral replication but this SNP was not above the limit of detection in S-ExoN1 P10'. We observed marked bias for T-to-C and A-to-G transition mutations in Sanger and deep sequencing data from S-ExoN1 (Table S7). These biases are consistent with the misincorporation tendency of RdRps [46]. Only two deletions (of three or 30 nt) were identified in this study, and no deletions and only one insertion was found in our MHV study [20]. Since deletions or insertions were not more or less frequent in ExoN mutant than WT viruses, this suggests that the mechanisms such as RNA recombination and RdRp stuttering or slippage underlying these processes are unaltered by disruption of ExoN, although further analysis is required to strengthen this argument. While we predict that each of the mutations identified in P3 clones and the majority of those identified in S-ExoN1 P1', P5', and P10' populations have little or no effect on viral growth, this prediction must be confirmed experimentally.

### Concluding remarks

Concordant results with MHV and SARS-CoV ExoN mutants support the theory that nsp14-ExoN is involved in RNA proofreading among group 2 coronaviruses and perhaps all coronaviruses, and that acquisition of ExoN activity was essential for attainment and maintenance of large nidovirus genomes. The present study further demonstrates that the ExoN mutator phenotype is stable over passage, generates increased population diversity over passage, and results in the accumulation and retention of large numbers of novel replication-tolerated mutations and mutation sets in every sequenced genome. In this report, we identified 100 mutations in 10 progeny of a single parental virus after limited replication cycles. The results suggest that S-ExoN1 may allow the identification of comprehensive maps and frequencies of mutations and mutation sets across the genome that can be classified as neutral, complementing, or deleterious. In combination with similar approaches with MHV-ExoN mutant viruses, it may be possible to rapidly define genomic positions or regions that are tolerant or intolerant of substitution in coronaviruses, which in turn could be tested as virus family-wide determinants of replication and pathogenesis. Finally, the differences in mutation frequency between WT and ExoN mutants of both SARS-CoV and MHV, in combination with the new bioinformatic methods for analysis of deep sequence data reported herein, may allow testing of the impact of altering the mutation-selection equilibrium on virus fitness, adaptation, or extinction in vitro and in vivo.

## Supporting Information

Figure S1 Distribution of mutations in individual viral clones across the genome. Mutations in individual viral clones are plotted according to position in the SARS-CoV genome (drawn to scale). Mutations in 10 SARS-WT (A) and 10 S-ExoN1 (B) P3 viral clones. Mutation types are indicated using the symbol and color scheme in Figure 5. Engineered ExoN1 mutations are depicted as bent vertical lines. Dotted vertical lines in panel B represent boundaries of coding regions (omitted for ORFs 8a and 9b for clarity).
(0.79 MB TIF)

Figure S2 Depth of coverage from deep sequencing. For each nucleotide position in the SARS-CoV genome the combined number of forward and reverse reads is plotted for SARS-WT P10′. Patterns of sequencing coverage were similar for the other five samples. Locations of the 13 amplicons subjected to sequencing are depicted by red boxes for comparison with the coverage data.
(0.26 MB TIF)

Figure S3 Frequencies of SNPs at P1′, P5′, and P10′. (A) Frequencies of the 12 SNPs identified in SARS-WT and detailed in Table S5 are shown for P1′, P5′, and P10′. (B) Frequencies of the 68 SNPs identified in S-ExoN1 and detailed in are shown for P1′, P5′, and P10′. SNPs are ranked by nucleotide position. SNP frequency (proportion of reads) was determined by dividing the sum of forward and reverse reads containing a particular SNP by the sum of forward and reverse reads spanning the relevant position. Only dominant SNPs are shown, and SNP frequencies <0.05 (dashed line) were not plotted.
(1.79 MB TIF)

Table S1 Nucleotides sequenced by the Sanger method and accession numbers.
(0.05 MB PDF)

Table S2 Non-engineered mutations identified in SARS-WT viruses.
(0.06 MB PDF)

Table S3 Non-engineered mutations identified in S-ExoN1 viruses.
(0.09 MB PDF)

Table S4 Non-engineered mutations identified in S-ExoN1 P1 c1.
(0.05 MB PDF)

Table S5 SNPs identified in SARS-WT viruses at P1′, P5′, and P10′ by deep sequencing.
(0.06 MB PDF)

Table S6 SNPs identified in S-ExoN1 viruses at P1′, P5′, and P10′ by deep sequencing.
(0.09 MB PDF)

Table S7 Matrix of specific substitution types in S-ExoN1 Sanger and deep genomes.
(0.05 MB PDF)
