# Supplementary figures and images for: Infidelity of SARS-CoV Nsp14-Exonuclease Mutant Virus Replication Is Revealed by Complete Genome Sequencing
Source: PLoS Pathog. 2010 May 6;6(5):e1000896. doi: 10.1371/journal.ppat.1000896 (PMC2865531; doi:10.1371/journal.ppat.1000896)

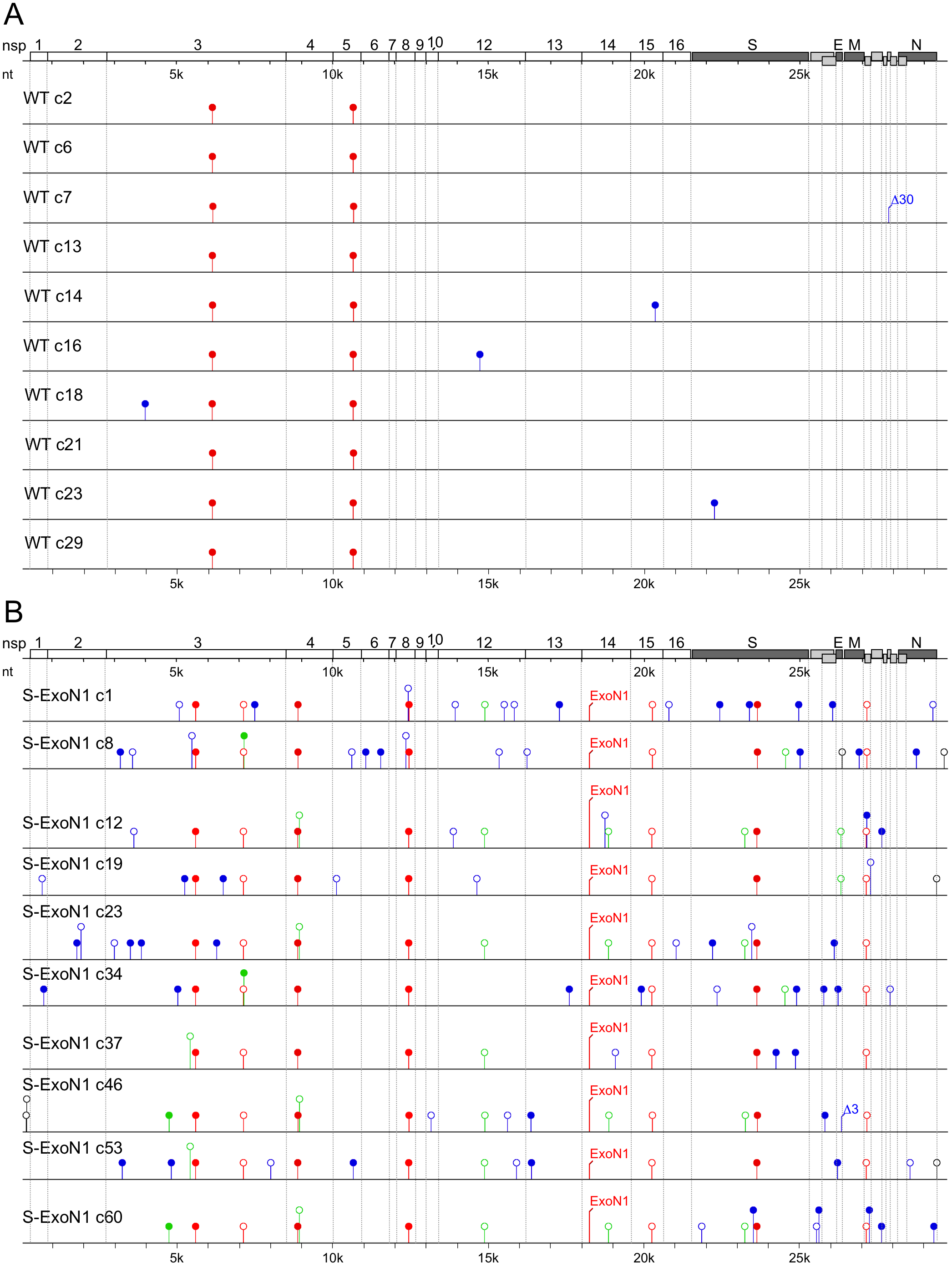

Supplement: Figure S1 — Distribution of mutations in individual viral clones across the genome. Mutations in individual viral clones are plotted according to position in the SARS-CoV genome (drawn to scale). Mutations in 10 SARS-WT (A) and 10 S-ExoN1 (B) P3 viral clones. Mutation types are indicated using the symbol and color scheme in Figure 5. Engineered ExoN1 mutations are depicted as bent vertical lines. Dotted vertical lines in panel B represent boundaries of coding regions (omitted for ORFs 8a and 9b for clarity). (0.79 MB TIF) [file ppat.1000896.s001.tif]

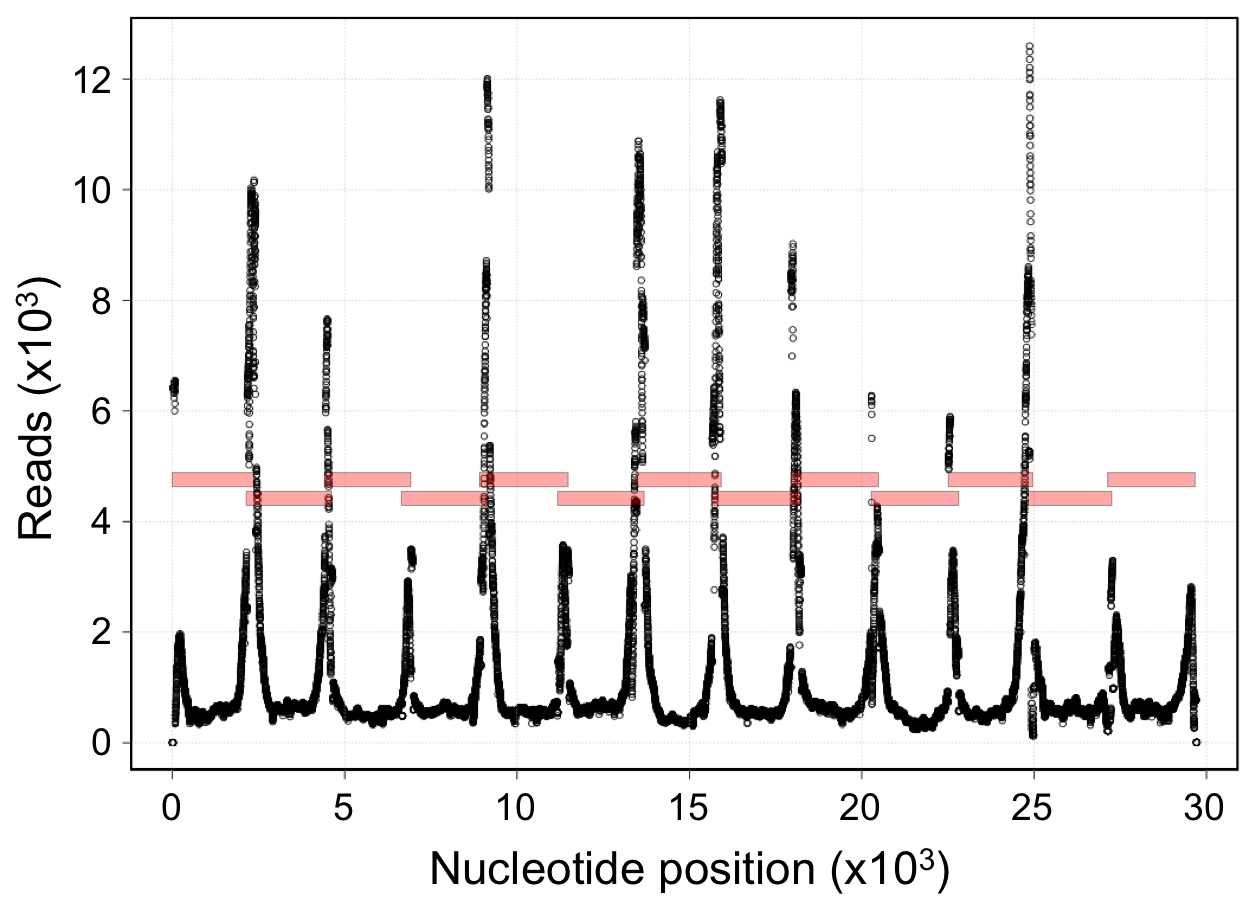

Supplement: Figure S2 — Depth of coverage from deep sequencing. For each nucleotide position in the SARS-CoV genome the combined number of forward and reverse reads is plotted for SARS-WT P10′. Patterns of sequencing coverage were similar for the other five samples. Locations of the 13 amplicons subjected to sequencing are depicted by red boxes for comparison with the coverage data. (0.26 MB TIF) [file ppat.1000896.s002.tif]

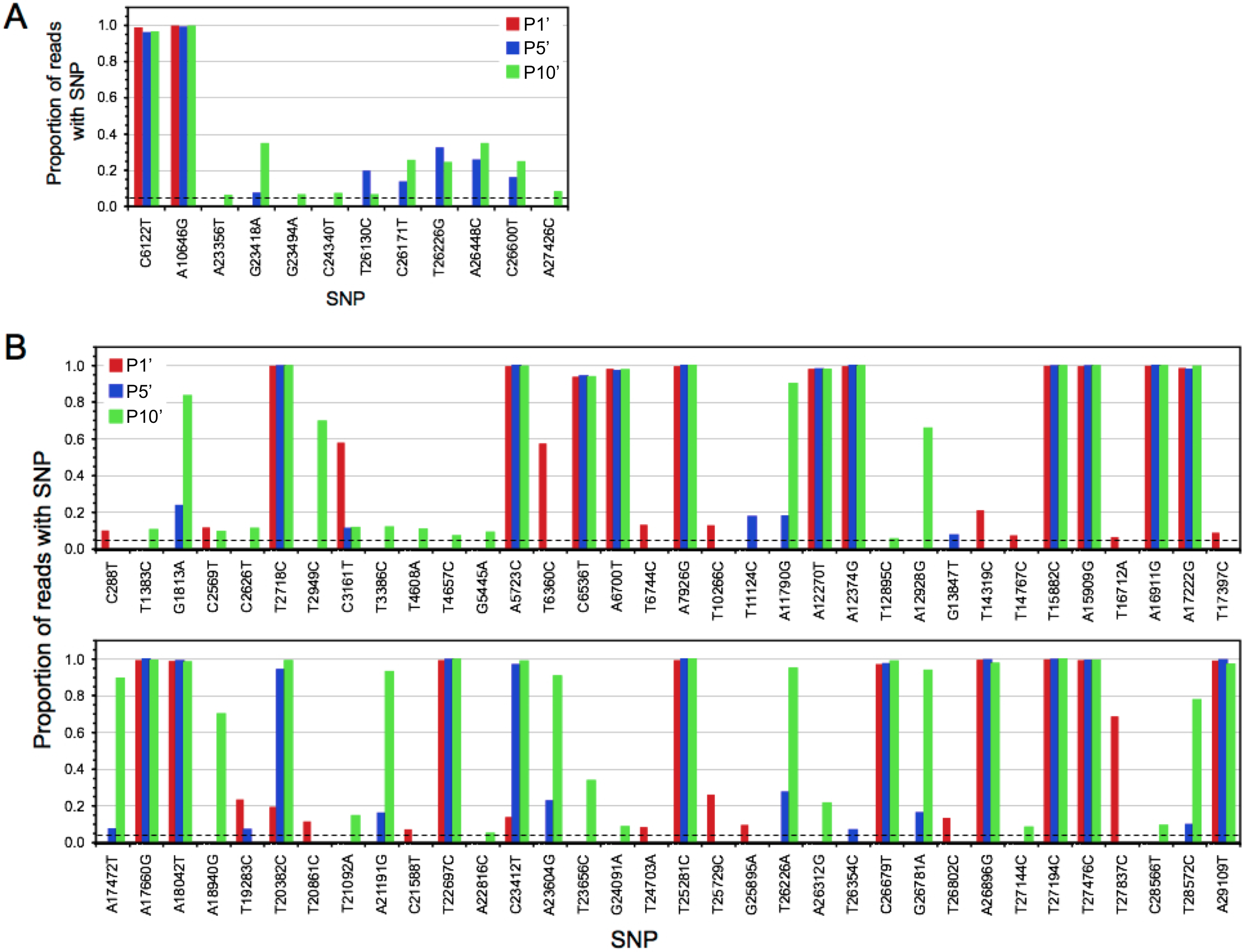

Supplement: Figure S3 — Frequencies of SNPs at P1′, P5′, and P10′. (A) Frequencies of the 12 SNPs identified in SARS-WT and detailed in Table S5 are shown for P1′, P5′, and P10′. (B) Frequencies of the 68 SNPs identified in S-ExoN1 and detailed in are shown for P1′, P5′, and P10′. SNPs are ranked by nucleotide position. SNP frequency (proportion of reads) was determined by dividing the sum of forward and reverse reads containing a particular SNP by the sum of forward and reverse reads spanning the relevant position. Only dominant SNPs are shown, and SNP frequencies <0.05 (dashed line) were not plotted. (1.79 MB TIF) [file ppat.1000896.s003.tif]
