# Supplementary material for: Infidelity of SARS-CoV Nsp14-Exonuclease Mutant Virus Replication Is Revealed by Complete Genome Sequencing
Source: PLoS Pathog. 2010 May 6;6(5):e1000896. doi: 10.1371/journal.ppat.1000896 (PMC2865531; doi:10.1371/journal.ppat.1000896)
Supplement: Table S1 — Nucleotides sequenced by the Sanger method and accession numbers. (0.05 MB PDF) [file ppat.1000896.s004.pdf]

Table S1. Nucleotides sequenced by the Sanger method and accession numbers.

Virus	Stock[a]	No. of nucleotides sequenced[b]	Region sequenced	GenBank accession no.
SARS-WT	P0 pop.	29687	21-29707	FJ882938
	P1 c1	29687	21-29707	FJ882927
	P3 c2	29646	41-29686	FJ882936
	P3 c6	29648	39-29686	FJ882933
	P3 c7	29646	41-29686	FJ882947
	P3 c13	29647	40-29686	FJ882946
	P3 c14	29646	41-29686	FJ882932
	P3 c16	29646	41-29686	FJ882939
	P3 c18	29648	39-29686	FJ882937
	P3 c21	29647	40-29686	FJ882935
	P3 c23	29647	40-29686	FJ882949
	P3 c29	29647	41-29687	FJ882934
S-ExoN1	P0 pop. #3	29687	21-29707	FJ882930
	P0 pop. #4	29675	33-29707	FJ882926
	P1 c1	29687	21-29707	FJ882928
	P3 c1	29665	22-29686	FJ882929
	P3 c8	29665	22-29686	FJ882941
	P3 c12	29646	41-29686	FJ882931
	P3 c19	29646	41-29686	FJ882955
	P3 c23	29628	59-29686	FJ882944
	P3 c34	29646	41-29686	FJ882960
	P3 c37	29646	41-29686	FJ882940
	P3 c46	29639	48-29686	FJ882954
	P3 c53	29644	41-29684	FJ882956
	P3 c60	29646	41-29686	FJ882950

[a] pop., population virus stock. S-ExoN1 P0 pop. #3 and #4 were derived from independent electroporation and virus recovery experiments.

[b] The complete SARS-CoV Urbani genome is 29727 nt, excluding the 3' poly(A) tail. Twenty-five genome sequences totaling 741,362 nt were determined by the Sanger method in this study.
