# Supplementary material for: Infidelity of SARS-CoV Nsp14-Exonuclease Mutant Virus Replication Is Revealed by Complete Genome Sequencing
Source: PLoS Pathog. 2010 May 6;6(5):e1000896. doi: 10.1371/journal.ppat.1000896 (PMC2865531; doi:10.1371/journal.ppat.1000896)
Supplement: Table S2 — Non-engineered mutations identified in SARS-WT viruses. (0.06 MB PDF) [file ppat.1000896.s005.pdf]

Table S2. Non-engineered mutations identified in SARS-WT viruses.

P3 viral clone[a]	Mutation[b,c]	Codon change	Amino acid change[f]	Location
All 10	C 6122 T	TCA→TTA	Ser 1135 Leu	nsp 3
	A 10646 G[d]	AAT→AGT	Asn 221 Ser	nsp 5
7	Δ27856-27885[e]	Multiple	Internal deletion	ORF 8a
		Multiple	Late start	ORF 8b
14	C 20332 T	TCA→TTA	Ser 261 Leu	nsp 15
16	G 14715 T	GCT→TCT	Ala 449 Ser	nsp 12
18	A 3971 C	AAT→ACT	Asn 418 Thr	nsp 3
23	G 22251 T	GTT→TTT	Val 254 Phe	Spike

[a] The 10 P3 clones analyzed were c2, c6, c7, c13, c14, c16, c18, c21, c23, and c29, and all were derived from parental clone P1 c1 that had only C6122T and A10646G mutations. Entire genomes were sequenced except for the terminal nucleotides as described in Table S1.

[b] Nucleotide positions refer to SARS-CoV Urbani complete genome sequence (GenBank accession no. AY278741).

[c] Red, mutations in P1 c1 and all 10 P3 clones; blue, mutations unique to single P3 clones.

[d] P0 population virus had subpopulations containing A or G at nt 10646.

[e] P3 c7 had a 30-nt deletion of nt 27856-27885 that removes 10 internal codons from ORF 8a and the first 22 nt, including the normal start codon, of ORF 8b. Since the next potential start codon for ORF 8b is 35 codons downstream, the deletion effectively removes the amino-terminal 35 aa of protein 8b.

[f] Amino acid positions in nsps refer to location within the respective mature nsp.
