# Supplementary material for: Infidelity of SARS-CoV Nsp14-Exonuclease Mutant Virus Replication Is Revealed by Complete Genome Sequencing
Source: PLoS Pathog. 2010 May 6;6(5):e1000896. doi: 10.1371/journal.ppat.1000896 (PMC2865531; doi:10.1371/journal.ppat.1000896)
Supplement: Table S3 — Non-engineered mutations identified in S-ExoN1 viruses. (0.09 MB PDF) [file ppat.1000896.s006.pdf]

Table S3. Non-engineered mutations identified in S-ExoN1 viruses.

P3 viral clone(s)[a]	Mutation[b]	Codon change	Amino acid change[d]	Location[i]
All 10	T 5588 C	GTA→GCA	Val 957 Ala	nsp 3
	G 7125 A	ACG→ACA	none	nsp 3
	G 8872 A	GGC→AGC	Gly 130 Ser	nsp 4
	A 12439 T	AAC→TAC	Asn 140 Tyr	nsp 8
	T 20258 C	GTT→GTC	none	nsp 15
	G 23631 A	GCT→ACT	Ala 714 Thr	Spike
	T 27148 C	GCT→GCC	none	ORF 6
46, 60	T 4731 A	GAT→GAA	Asp 671 Glu	nsp 3
37, 53	T 5412 C	GCT→GCC	none	nsp 3
8, 34	T 7142 C	CTA→CCA	Leu 1475 Pro	nsp 3
12, 23, 46, 60	T 8922 C	GCT→GCC	none	nsp 4
1, 12, 23, 37, 46, 53, 60	T 14876 A	GCT→GCA	none	nsp 12
12, 23, 46, 60	T 18860 C	CCT→CCC	none	nsp 14
12, 23, 46, 60	T 23243 C	ATT→ATC	none	Spike
8, 34	T 24536 C	GTT→GTC	none	Spike
12, 19	T 26335 C	GAT→GAC	none	E
1	T 5061 C	CCT→CCC	none	nsp 3
	T 7484 G	ATG→AGG	Met 1589 Arg	nsp 3
	T 12411 C	GTT→GTC	none	nsp 8
	A 13925 G	TCA→TCG	none	nsp 12
	A 15497 G	TCA→TCG	none	nsp 12
	A 15821 T	ACA→ACT	none	nsp 12
	C 17269 T	GCT→GTT	Ala 368 Val	nsp 13
	T 20789 C	GTT→GTC	none	nsp 16
	G 22423 T	GGA→GTA	Gly 311 Val	Spike (RBD)
	T 23374 C	GTA→GCA	Val 628 Ala	Spike
	G 24957 A	GCT→ACT	Ala 1156 Thr	Spike
	C 26049 T	CCA→CTA	Pro 261 Leu	ORF 3a
		CAA→TAA	Gln 121 Stop[e]	ORF 3b
	G 29271 A	CCG→CCA	none	N
8	G 3166 A	GCT→ACT	Ala 150 Thr	nsp 3
	T 3558 C	CCT→CCC	none	nsp 3
	G 5466 A	CAG→CAA	none	nsp 3
	A 10602 G	GCA→GCG	none	nsp 5
	A 11050 G	ATG→GTG	Met 50 Val	nsp 6
	A 11530 G	ATC→GTC	Ile 210 Val	nsp 6
	T 12339 C	ATT→ATC	none	nsp 8
	T 15333 C	TTA→CTA	none	nsp 12
	A 16232 G	AGA→AGG	none	nsp 13
	G 25002 A	GTC→ATC	Val 1171 Ile	Spike
	T 26354 C	n.a.	n.a.	IGR
	A 26899 T	ACT→TCT	Thr 168 Ser	M
	A 28736 T	AAT→ATT	Asn 206 Ile	N
	A 29628 G	n.a.	n.a.	3' UTR
12	T 3603 C	TAT→TAC	none	nsp 3
	T 13874 C	AAT→AAC	none	nsp 12
	T 18749 C	CAT→CAC	none	nsp 14
	A 27167 G	ATA→GTA	Ile 32 Val	ORF 6
	T 27648 C	CTC→CCC	Leu 4 Pro	ORF 7b
19	A 654 T	GGA→GGT	none	nsp 1
	T 5236 A	TTA→ATA	Leu 840 Ile	nsp 3
	A 6476 G	GAT→GGT	Asp 1253 Gly	nsp 3
	A 10119 G	ACA→ACG	none	nsp 5
	T 14630 C	TAT→TAC	none	nsp 12
	C 27290 T	TTC→TTT	none	ORF 7a
	C 29412 T	n.a.	n.a.	3' UTR
23	G 1771 A	GTT→ATT	Val 323 Ile	nsp 2
	T 1905 C	ATT→ATC	none	nsp 2
	T 2976 C	GCT→GCC	none	nsp 3
	C 3491 T	ACA→ATA	Thr 258 Ile	nsp 3
	A 3845 G	GAG→GGG	Glu 376 Gly	nsp 3

	T 6265 C	TCA→CCA	Ser 1183 Pro	nsp 3
	C 21038 T	TTC→TTT	none	nsp 16
	T 22206 C	TCA→CCA	Ser 239 Pro	Spike
	T 23468 C	AGT→AGC	none	Spike
	A 26121 T	TAC→TTC	Tyr 2 Phe	E
		ACT→TCT	Thr 145 Ser	ORF 3b
34	A 707 G	GAG→GGG	Glu 148 Gly	nsp 1
	T 5015 C	ATT→ACT	Ile 766 Thr	nsp 3
	T 17602 C	GTT→GCT	Val 479 Ala	nsp 13
	T 19917 C	TTT→CTT	Phe 123 Leu	nsp 15
	A 22352 G	AAA→AAG	none	Spike
	A 24910 T	AAT→ATT	Asn 1140 Ile	Spike
	T 25783 A	GGT→GGA	none	ORF 3a
		GTG→GAG	Val 32 Glu	ORF 3b
	A 26241 G	TAC→TGC	Tyr 42 Cys	E
	T 27917 A	ACT→ACA	none	ORF 8b
37	T 19082 C	GCT→GCC	none	nsp 14
	C 24249 T	CTT→TTT	Leu 920 Phe	Spike
	G 24873 A	GAC→AAC	Asp 1128 Asn	Spike
46	T 141 C	n.a.	n.a.	5' UTR
	G 159 A	n.a.	n.a.	5' UTR
	A 13149 G	CAA→CAG	none	nsp 10
	T 15605 C	TTT→TTC	none	nsp 12
	T 16356 C	TAT→CAT	Tyr 64 His	nsp 13
	C 25800 T	CCA→CTA	Pro 178 Leu	ORF 3a
		CAA→TAA	Gln 38 Stop[f]	ORF 3b
	Δ_{26340}TGG$_{26342}$[c]	C(TG.G)TC →CTC	ΔVal 76	E
53	T 3229 C	TCA→CCA	Ser 171 Pro	nsp 3
	T 4808 C	CTG→CCG	Leu 697 Pro	nsp 3
	T 8004 C	AGT→AGC	none	nsp 3
	C 10658 T	ACT→ATT	Thr 225 Ile	nsp 5
	T 15905 C	GAT→GAC	none	nsp 12
	T 16386 C	TCA→CCA	Ser 74 Pro	nsp 13
	T 26226 A	CTT→CAT	Leu 37 His	E
	C 28557 T	CAC→CAT	none	N
	G 29417 A	n.a.	n.a.	3' UTR
60	T 21860 A	GTT→GTA	none	Spike
	C 23518 T	GCT→GTT	Ala 676 Val	Spike
	T 25550 C	TTG→CTG	none	ORF 3a
	C 25626 T	GCA→GTA	Ala 120 Val	ORF 3a
	G 27248 A	GAG→AAG	Glu 59 Lys	ORF 6
	T 27639 C	TGA→CGA	Stop→Arg[g]	ORF 7a
		ATG→ACG	Met 1 Thr[h]	ORF 7b
	A 29324 G	GAT→GGT	Asp 402 Gly	N

[a] P3 clones analyzed were c1, c8, c12, c19, c23, c34, c37, c46, c53, and c60. Entire genomes were sequenced except for terminal nucleotides as indicated in Table S1.

[b] Red, mutations in all clones; green, mutations in a subset of clones, blue, mutations unique to single clones.

[c] We cannot differentiate between Δ_{26340}TGG$_{26342}$ [C(TG.G)TC→CTC] or Δ_{26341}GGT$_{26343}$ [CT(G.GT)C→CTC], but both have the same effect of deleting the C-terminal Val76 residue of E protein.

[d] Amino acid positions in nsps refer to location within the respective mature nsp. 'None' indicates synonymous changes. Note that the Δ_{26340}TGG$_{26342}$ mutation in c46 removes nucleotides from adjacent codons.

[e] Early termination removes the carboxy-terminal 34 residues of the ORF 3b protein.

[f] Early termination removes the carboxy -terminal 117 residues of the ORF 3b protein.

[g] Effectively adds five residues (RMSSL) to the C-terminus of the ORF 7a protein.

[h] Effectively removes the 23 amino-terminal residues from the ORF 7b protein.

[i] IGR, intergenic region; UTR, untranslated region at either terminus of the genome; RBD, receptor-binding domain.
