# Supplementary material for: Infidelity of SARS-CoV Nsp14-Exonuclease Mutant Virus Replication Is Revealed by Complete Genome Sequencing
Source: PLoS Pathog. 2010 May 6;6(5):e1000896. doi: 10.1371/journal.ppat.1000896 (PMC2865531; doi:10.1371/journal.ppat.1000896)
Supplement: Table S4 — Non-engineered mutations identified in S-ExoN1 P1 c1. (0.05 MB PDF) [file ppat.1000896.s007.pdf]

Table S4. Non-engineered mutations identified in S-ExoN1 P1 c1.

Mutation[a]	Codon change	Amino acid change[b]	Location
C 4382 T	GCC→GTC	Ala 555 Val	nsp 3
T 6494 A	GTG→GAG	Val 1259 Glu	nsp 3
T 17465 C	GGT→GGC	none	nsp 13
C 20288 T	GGC→GGT	none	nsp 15
C 25355 T	CAT→TAT	His 30 Tyr	ORF 3a
C 29454 T	n.a.	n.a.	3' UTR

[a] The entire genome was sequenced except for the terminal 20 nt at each end.

[b] Amino acid positions in nsps refer to location within the respective mature nsp. 'None' indicates synonymous changes.
