# Supplementary material for: Infidelity of SARS-CoV Nsp14-Exonuclease Mutant Virus Replication Is Revealed by Complete Genome Sequencing
Source: PLoS Pathog. 2010 May 6;6(5):e1000896. doi: 10.1371/journal.ppat.1000896 (PMC2865531; doi:10.1371/journal.ppat.1000896)
Supplement: Table S5 — SNPs identified in SARS-WT viruses at P1′, P5′, and P10′ by deep sequencing. (0.06 MB PDF) [file ppat.1000896.s008.pdf]

Table S5. SNPs identified in SARS-WT viruses at P1', P5', and P10' by deep sequencing.

Passage	Mutation[a]	Codon change	Amino acid change[b]	Location	SNP frequency[c] P1'	P5'	P10'
1', 5', 10'	C 6122 T	TCA→TTA	Ser 1135 Leu	nsp 3	0.9908	0.9645	0.9685
	A 10646 G	AAT→AGT	Asn 221 Ser	nsp 5	1.0000	0.9957	1.0000
5', 10'	G 23418 A	GAC→AAC	Asp 643 Asn	Spike		0.0773	0.3522
	T 26130 C	TTT→CTT	Phe 148 Leu	ORF 3b		0.2006	0.0681
		GTT→GCT	Val 5 Ala	E			
	C 26171 T	CTT→TTT	Leu 19 Phe	E		0.1405	0.2591
	T 26226 G	CTT→CGT	Leu 37 Arg	E		0.3275	0.2473
	A 26448 C	GAA→GAC	Glu 17 Asp	M		0.2617	0.3511
	C 26600 T	GCT→GTT	Ala 68 Val	M		0.1633	0.2528
10'	A 23356 T	TAT→TTT	Tyr 622 Phe	Spike			0.0675
	G 23494 A	AGT→AAT	Ser 668 Asn	Spike			0.0694
	C 24340 T	TCT→TTT	Ser 950 Phe	Spike			0.0753
	A 27426 C	AAT→CAT	Asn 52 His	ORF 7a			0.0846

[a] Red, mutations in all 3 passages; green, shared in P5' and P10' but not P1'; blue, unique to a single passage.
[b] Amino acid positions within nsps refer to location within the respective mature nsp.
[c] Proportion of reads containing each SNP at the passage indicated. SNP frequencies of ≥0.05 are shown.
