# Supplementary material for: Infidelity of SARS-CoV Nsp14-Exonuclease Mutant Virus Replication Is Revealed by Complete Genome Sequencing
Source: PLoS Pathog. 2010 May 6;6(5):e1000896. doi: 10.1371/journal.ppat.1000896 (PMC2865531; doi:10.1371/journal.ppat.1000896)
Supplement: Table S6 — SNPs identified in S-ExoN1 viruses at P1′, P5′, and P10′ by deep sequencing. (0.09 MB PDF) [file ppat.1000896.s009.pdf]

Table S6. SNPs identified in S-ExoN1 viruses at P1’, P5’, and P10’ by deep sequencing.

Passage	Mutation[a]	Codon change	Amino acid change[b]	Location[c]	SNP frequency[d]		
					P1’	P5’	P10’
1’, 5’, 10’	T 2718 C	GGT→GGC	none	nsp 2	1	0.9969	0.9971
	C 3161 T	GCC→GTC	Ala 148 Val	nsp 3	0.5815	0.1126	0.1174
	A 5723 C	GAG→GCG	Glu 1002 Ala	nsp 3	0.9976	1	0.9949
	C 6536 T	TCA→TTA	Ser 1273 Leu	nsp 3	0.9398	0.9429	0.9383
	A 6700 T	ATG→TTG	Met 1328 Leu	nsp 3	0.9836	0.9713	0.9757
	A 7926 G	GTA→GTG	none	nsp 3	0.998	1	1
	A 12270 T	GTA→GTT	none	nsp 8	0.9838	0.98	0.9779
	A 12374 G	AAC→AGC	Asn 118 Ser	nsp 8	0.9983	1	0.9963
	T 15882 C	TTA→CTA	none	nsp 12	0.9984	0.9974	0.998
	A 15909 G	ATT→GTT	Ile 847 Val	nsp 12	0.9979	0.9971	0.9982
	A 16911 G	ATT→GTT	Ile 249 Val	nsp 13	1	1	0.9972
	A 17222 G	CTA→CTG	none	nsp 13	0.9879	0.979	0.9957
	A 17660 G	GAA→GAG	none	nsp 13	0.9956	1	0.9933
	A 18042 T	ACA→TCA	Thr 25 Ser	nsp 14	0.989	0.989	0.9817
	T 20382 C	TAC→CAC	Tyr 278 His	nsp 15	0.1951	0.9428	0.9897
	T 22697 C	ACT→ACC	none	Spike	0.9948	0.9965	0.997
	C 23412 T	CAT→TAT	His 641 Tyr	Spike	0.1412	0.969	0.9865
	T 25281 C	ATG→ACG	Met 5 Thr	ORF 3a	0.9953	1	1
	C 26679 T	TAC→TAT	none	M	0.973	0.974	0.9876
	A 26896 G	ATC→GTC	Ile 167 Val	M	0.9974	0.9957	0.976
	T 27194 C	TTT→CTT	Phe 41 Leu	ORF 6	1	0.9982	1
	T 27476 C	GCT→GCC	none	ORF 7a	0.9957	0.9914	0.9924
	A 29109 T	ACA→ACT	none	N	0.9919	0.9955	0.9705
1’, 5’	T 19283 C	AAT→AAC	none	nsp 14	0.2378	0.0727	
1’, 10’	C 2569 T	CCC→TCC	Pro 589 Ser	nsp 2	0.12		0.0958
5’, 10’	G 1813 A	GTA→ATA	Val 337 Ile	nsp 2		0.2362	0.8339
	A 11790 G	GTA→GTG	none	nsp 7		0.1788	0.9016
	A 17472 T	ATG→TTG	Met 436 Leu	nsp 13		0.0741	0.8947
	A 21191 G	TCA→TCG	none	nsp 16		0.1603	0.9291
	A 23604 G	ACT→GCT	Thr 705 Ala	Spike		0.2272	0.9069
	T 26226 A	CTT→CAT	Leu 37 His	E		0.276	0.9492
	G 26781 A	GTG→GTA	none	M		0.1622	0.9375
	T 28572 C	AAT→AAC	none	N		0.0977	0.7783
1’	C 288 T	GTC→GTT	none	nsp 1	0.1036		
	T 6360 C	CCT→CCC	none	nsp 3	0.5773		
	T 6744 C	ACT→ACC	none	nsp 3	0.1338		
	T 10266 C	TCT→TCC	none	nsp 5	0.1309		
	T 14319 C	TTT→CTT	Phe 317 Leu	nsp 12	0.2129		
	T 14767 C	ATC→ACC	Ile 466 Thr	nsp 12	0.0762		
	T 16712 A	TTT→TTA	Phe 182 Leu	nsp 13	0.0671		
	T 17397 C	TTG→CTG	none	nsp 13	0.09		
	T 20861 C	ACT→ACC	none	nsp 16	0.1169		
	C 21588 T	CAT→TAT	His 33 Tyr	Spike	0.0737		
	T 24703 A	TTC→TAC	Phe 1071 Tyr	Spike	0.0864		
	T 25729 C	TAT→TAC	none	ORF 3a	0.2634		
		ATG→ACG	Met 14 Thr	ORF 3b			
	G 25895 A	GTT→ATT	Val 210 Ile	ORF 3a	0.0975		
		AAG→AAA	none	ORF 3b			
	T 26802 C	AGT→AGC	none	M	0.1366		
	T 27837 C	GTA→GCA	Val 20 Ala	ORF 8a	0.6885		
5’	T 11124 C	TCT→TCC	none	nsp 6		0.1765	
	G 13847 T	AAG→AAT	Lys 159 Asn	nsp 12		0.0775	
	T 26354 C	n.a.	n.a.	IGR		0.0685	
10’	T 1383 C	TGT→TGC	none	nsp 2			0.1061
	C 2626 T	CTC→TTC	Leu 608 Phe	nsp 2			0.112
	T 2949 C	GGT→GGC	none	nsp 3			0.6963
	T 3386 C	ATA→ACA	Ile 223 Thr	nsp 3			0.1189
	T 4608 A	CCT→CCA	none	nsp 3			0.1072
	T 4657 C	TAC→CAC	Tyr 647 His	nsp 3			0.0706
	G 5445 A	GTG→GTA	none	nsp 3			0.0908
	T 12895 C	TTA→CTA	none	nsp 9			0.0552
	A 12928 G	AGT→GGT	Ser 105 Gly	nsp 9			0.6579

A 18940 G	GAT→GGT	Asp 324 Gly	nsp 14	0.7004
T 21092 A	GCT→GCA	none	nsp 16	0.1457
A 22816 C	TAT→TCT	Tyr 442 Ser	Spike RBM	0.0502
T 23656 C	ATG→ACG	Met 722 Thr	Spike	0.3364
G 24091 A	GGA→GAA	Gly 867 Glu	Spike	0.0856
A 26312 G	AAC→GAC	Asn 66 Asp	E	0.2145
T 27144 C	ATT→ACT	Ile 24 Thr	ORF 6	0.0847
C 28566 T	ACC→ACT	none	N	0.0935

[a] Red, mutations in all 3 passages; purple, shared in P1' and P5' but not P10'; orange, shared in P1' and P10' but not P5'; green, shared in P5' and P10' but not P1'; blue, mutations unique to a single passage.
[b] Amino acid positions within nsps refer to location within the respective mature nsp. 'None' indicates synonymous changes.
[c] IGR, intergenic region; RBM, receptor binding motif.
[d] Proportion of reads containing each SNP at the passage indicated. SNP frequencies of ≥0.05 are shown.
