# Supplementary material for: Infidelity of SARS-CoV Nsp14-Exonuclease Mutant Virus Replication Is Revealed by Complete Genome Sequencing
Source: PLoS Pathog. 2010 May 6;6(5):e1000896. doi: 10.1371/journal.ppat.1000896 (PMC2865531; doi:10.1371/journal.ppat.1000896)
Supplement: Table S7 — Matrix of specific substitution types in S-ExoN1 Sanger and deep genomes. (0.05 MB PDF) [file ppat.1000896.s010.pdf]

Table S7. Matrix of specific substitution types in S-ExoN1 Sanger and deep genomes.

WT nt	Substitution[a]			
	A	C	G	T
A	–	0 (2.9)	16.1 (19.1)	7.1 (7.4)
C	0 (0)	–	0 (0)	12.1 (13.2)
G	13.1 (7.4)	0 (0)	–	1.0 (1.5)
T	7.1 (7.4)	42.4 (41.2)	1.0 (0)	–

[a] Percentages of total non-redundant substitutions in 10 combined S-ExoN1 P3 clones determined by Sanger sequencing are shown, and those in combined P1', P5', and P10' S-ExoN1 determined by deep sequencing are shown in parentheses. Blue, transitions; black, transversions.
